# Gravity models for potential spatial healthcare access measurement: a systematic methodological review

**DOI:** 10.1186/s12942-023-00358-z

**Published:** 2023-12-01

**Authors:** Barbara Stacherl, Odile Sauzet

**Affiliations:** 1 Socio-Economic Panel (SOEP), German Institute for Economic Research (DIW Berlin), Mohrenstraße 58, 11017 Berlin, Germany; 2 School of Public Health, Bielefeld University, Universitätsstraße 25, 33615 Bielefeld, Germany; 3 Department of Business Administration and Economics, Bielefeld University, Universitätsstraße 25, 33615 Bielefeld, Germany

**Keywords:** Gravity models, 2SFCA, Kernel density, Methodological review, Potential spatial access, Healthcare, Spatial access measurement

## Abstract

**Background:**

Quantifying spatial access to care—the interplay of accessibility and availability—is vital for healthcare planning and understanding implications of services (mal-)distribution. A plethora of methods aims to measure potential spatial access to healthcare services. The current study conducts a systematic review to identify and assess gravity model-type methods for spatial healthcare access measurement and to summarize the use of these measures in empirical research.

**Methods:**

A two-step approach was used to identify (1) methodological studies that presented a novel gravity model for measuring spatial access to healthcare and (2) empirical studies that applied one of these methods in a healthcare context. The review was conducted according to the PRISMA guidelines. *EMBASE*, *CINAHL*, *Web of Science*, and *Scopus* were searched in the first step. Forward citation search was used in the second step.

**Results:**

We identified 43 studies presenting a methodological development and 346 empirical application cases of those methods in 309 studies. Two major conceptual developments emerged: The Two-Step Floating Catchment Area (2SFCA) method and the Kernel Density (KD) method. Virtually all other methodological developments evolved from the 2SFCA method, forming the 2SFCA method family. Novel methodologies within the 2SFCA family introduced developments regarding distance decay within the catchment area, variable catchment area sizes, outcome unit, provider competition, local and global distance decay, subgroup-specific access, multiple transportation modes, and time-dependent access. Methodological developments aimed to either approximate reality, fit a specific context, or correct methodology. Empirical studies almost exclusively applied methods from the 2SFCA family while other gravity model types were applied rarely. Distance decay within catchment areas was frequently implemented in application studies, however, the initial 2SFCA method remains common in empirical research. Most empirical studies used the spatial access measure for descriptive purposes. Increasingly, gravity model measures also served as potential explanatory factor for health outcomes.

**Conclusions:**

Gravity models for measuring potential spatial healthcare access are almost exclusively dominated by the family of 2SFCA methods—both for methodological developments and applications in empirical research. While methodological developments incorporate increasing methodological complexity, research practice largely applies gravity models with straightforward intuition and moderate data and computational requirements.

**Supplementary Information:**

The online version contains supplementary material available at 10.1186/s12942-023-00358-z.

## Introduction

Access to healthcare is an important determinant of health outcomes [1, 2]. Ensuring adequate access is therefore a central goal of health systems globally [3]. As part of that, achieving equitable geographic distribution of healthcare services—that is, spatial healthcare access—is a public health priority. Due to the spatial configuration of health systems, i.e., fixed and limited locations of health facilities serving unevenly and continuously distributed populations, there are inherent disparities in spatial access to healthcare [4]. The extent to which these disparities manifest depends on the allocation of healthcare resources within a health system [5]. Against this background, quantifying spatial healthcare access serves the important aims of describing disparities in spatial access, informing healthcare planning, and investigating effects on health outcomes. Consequently, sound measurement of spatial healthcare access is crucial for adequately addressing these aims.

While there is no uniform definition of “access” or “spatial access” to healthcare, there are generally accepted concepts of access that are frequently employed in health research. Penchansky and Thomas [6] described access as the “degree of fit” between healthcare providers and patients, identifying five distinct access dimensions: availability (provider resources; patient needs), accessibility (distance to provider; mobility), accommodation (organization of provider operation; perception of suitability), affordability (charges; ability to pay), and acceptability (practice characteristics; attitudes toward them). These dimensions were further categorized into aspatial (accommodation, affordability, acceptability) and spatial (availability, accessibility) components by Khan [7]. Importantly, in this conceptualization of spatial access, availability and accessibility interact rather than coexist. Lastly, potential and realized access were distinguished depending on whether actual utilization of healthcare (realized) is investigated, or characteristics of the healthcare system (potential) are of interest [4]. Drawing upon these established conceptualizations, the present research is concerned with potential spatial access.

Regarding the measurement of the spatial component of access, three general methodological approaches exist: (1) The spatial proximity approach quantifies travel costs, e.g., in terms of distance or travel time between provider and population locations. (2) The container approach quantifies the presence or density of providers within a predefined area. (3) The spatial interaction approach, or gravity model approach, quantifies both proximity and density of providers [8]. Thus, while spatial proximity focuses on accessibility and the container approach on availability, the gravity model approach integrates both. The interdependence of availability and accessibility is especially relevant in the healthcare context [7]. For example, the distance to the closest physician is only relevant if the practice has capacity to treat patients. For measuring potential spatial access as defined above, therefore, the gravity model approach is the most suitable [8].

The spatial dimension of health and healthcare has been on the minds of researchers and health policy makers for many years [9]. Relatedly, gravity model approaches for measuring potential spatial access to healthcare have a long history in the field of health geography [7, 10]. Joseph and Bantock [10] first proposed a gravity model which not only accounted for provider density and proximity to providers at the same time, but also integrated the demand intensity by adjusting for the population size. Almost two decades later, Luo and Wang [11] introduced another type of gravity model—the two-step floating catchment area (2SFCA) method. The 2SFCA method produces a local spatial access measure relating health services supply to population demand by using floating catchment areas centered around provider and population locations. Since then, many methodological developments to the 2SFCA method have been proposed, e.g., incorporating distance decay [12], variable catchment areas [13], or multiple transportation modes [14]. Gravity models in general and 2SFCA methods in particular have since become standard for potential spatial access measurement [9], being applied for describing spatial healthcare access (e.g., [15–17]), and evaluating effects on health outcomes (e.g., [18–20]). Previous efforts to summarize the literature on potential spatial access measurement include overviews of healthcare access measurement and 2SFCA developments [21, 22], comparative evaluation of modeling choice in gravity models [23, 24], as well as systematic reviews of empirical studies applying spatial access measures [25, 26].

Gravity models for healthcare access measurement have seen numerous developments over the years [21]. To date, however, no systematic methodological review has been conducted to comprehensively describe and assess these developments. Yet, to identify the method that best fits a research question, intuition, merits, and drawbacks of each method need to be evaluated. Relatedly, while gravity models are widely used in health research [9], it is not clear which of the methodological developments have caught on in practice and for which purposes are they applied.

The aim of the current study, therefore, is to conduct a systematic review of methodological developments and application studies on potential spatial healthcare access measurement. It is conducted in two stages. In the first step, methodological studies presenting novel developments of potential spatial healthcare access measurement will be identified, categorized by methodological properties, and assessed by purpose, complexity, and data requirements. In the second step, empirical studies applying one of these methods in the healthcare context will be summarized and characterized, illustrating to what degree the methodological developments are applied in research practice.

## Methods

We sought to identify (1) all studies that presented a methodological development of a gravity model for measuring spatial access to healthcare and (2) all empirical studies that applied one of these methodological developments in a healthcare context. This was done in two steps. Both review steps were conducted according to the PRISMA guidelines [27]. The PROSPERO registration details, and full review protocol are publicly available with the PROSPERO registration number CRD42022334001.

### Inclusion criteria

The goal was to identify methodological developments in step 1 and empirical applications in step 2. Our inclusion criteria concerning both steps were: Original articles of peer-reviewed journals; In the English language; Measuring spatial healthcare access; Using a gravity model integrating availability, accessibility, supply, and demand dimensions in one single measure. Studies were included only in a (human) healthcare context, i.e., articles investigating spatial access to food outlets, daycare facilities, or veterinary care facilities were excluded. In step 1, additionally, articles were included only if they presented a methodological development (detailed below). In step 2, articles were included only if they presented an empirical application of one of the methods identified in step 1.

To identify methodological developments (step 1), we used the method put forward by Joseph and Bantock [10] as a starting point. They were the first to propose a gravity model in the healthcare context that integrated demand (in addition to supply) factors. We refer to it as the base gravity model from here onward. It takes on the following form:

$${R}_{j}=\frac{{S}_{j}}{{\sum }_{i=1}^{n}{P}_{i}*{d}_{ij}^{-\beta }}$$

$${A}_{i}^{*}= {\sum }_{j=1}^{m}{R}_{j}*{d}_{ij}^{-\beta }$$

where ${S}_{j}$ is the healthcare capacity (= supply, e.g., number of pediatricians) at location $j$ (e.g., practice address), ${P}_{i}$ is the demand intensity (= population, e.g., inhabitants below age 15) at location $i$ (e.g., municipality centroid), ${d}_{ij}^{-\beta }$ is the distance decay function with ${d}_{ij}$ being the distance friction (e.g., travel time) between population and provider location and with $\beta$ being the distance friction parameter. ${R}_{j}$ is thus the provider-to-population ratio at location $j$ and ${A}_{i}^{*}$ is the final spatial access index at population location $i$.

Modifications of the above formulas were considered a methodological development. Studies that merely altered the ${S}_{j}$ (e.g., full-time equivalents instead of head counts), ${P}_{i}$ (e.g., health needs weighted population instead of general population), or ${d}_{ij}$ (e.g., routing distance instead of Euclidean distance) computation or plugged in different values for the friction parameter were not considered methodological developments.

### Search strategy

*Step 1:* To identify relevant literature, the medical literature databases *EMBASE* (including *MEDLINE*) and *CINAHL* as well as the multidisciplinary databases *Web of Science* and *Scopus* were systematically searched. We consulted survey papers and seminal articles in the field of spatial access measures to develop our search strategy [7, 8, 28, 29]. The final search cloud was refined through an iteration of searches. The search strategy for identification of articles was conducted using a combined search cloud within the title and abstract capturing the concepts gravity model, healthcare, spatial access, and indicator. The complete search strategy for step 1 is given in Additional file 1: Appendix A. Searches for literature identification in step 1 were conducted in May 2022.

*Step 2:* To identify empirical application studies, we used forward citation search yielding studies that cited one or more of the methodological developments from step 1. Forward citation search options are implemented in *Web of Science,* and *Scopus*, not, however, in *EMBASE,* and *CINAHL*, allowing us to use only two of the databases in step 2. Database searches were amended with studies that had been excluded in step 1 solely for not presenting a methodological development but fulfilling all other inclusion criteria. Studies that had been excluded in step 1 for other reasons (e.g., because no gravity model was presented) were automatically excluded from the step 2 search results. Searches for literature identification in step 2 were conducted in December 2022.

### Study selection

**Fig. 1:**
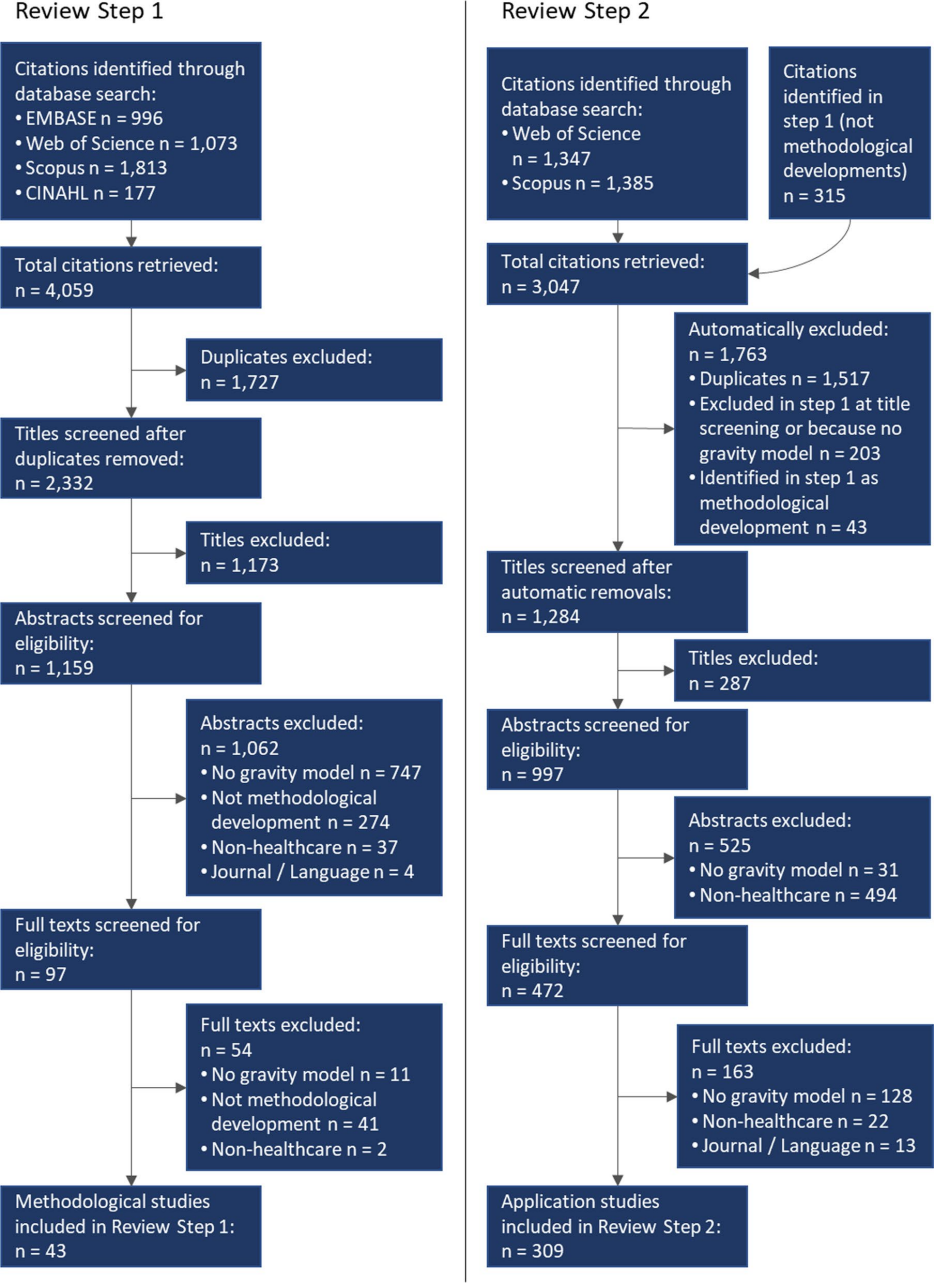
Study selection process

Abstracts of articles eligible for screening were first de-duplicated. Second, one reviewer (author one) conducted a title screening excluding titles indicating a non-healthcare context, non-suitable article type (e.g., review, editorial), or not written in English. Third, both reviewers screened the remaining article abstracts and full texts. During abstract and full text-screening for step 1, the reviewers were blinded to each other’s decision. Abstract and full text-screening to determine eligibility in step 2 was conducted by one reviewer (author one) and cross-checked by the other reviewer. Disagreements between regarding the inclusion of articles were resolved through discussion and consensus. The study selection process is depicted in Fig. 1.

### Data extraction and analysis

Data on study characteristics (title, authors, journal, year, and country of publication) and study background (study area, care sector) were independently extracted by one reviewer (author one). Methodological details were extracted and classified by both reviewers. In step 1, methodological development studies were classified into novelty categories. For each novelty category the specific methodological change, intuition, subsequent adaptations within the category, purpose, methodological complexity, and data requirements were assessed. In step 2, the application frequency for each novelty category was assessed by identifying the specific methodological developments used in each application study. Additionally, study characteristics of application studies were extracted. Lastly, we classified the purpose of the application study and determined whether the spatial healthcare access indicator was computed, or secondary data was used. With this approach we (1) characterized the gravity model-type methodological developments proposed for spatial access measurement in a healthcare context; and (2) synthesized how these models were applied in empirical research.

## Results

### Search results

In review step 1, 4059 citations were retrieved. After exclusion of 1727 duplicates, 1173 titles were excluded because they were out of the scope of this study. Upon abstract and full text-screening, 1116 citations were excluded because they either did not include a gravity model, were not in the field of healthcare, did not fit the journal/language criteria, or did not constitute a methodological development. We resulted with a final set of 43 studies that presented gravity model-type methodological developments for measuring spatial healthcare access (Additional file 1: Appendix B).

For review step 2, we retrieved 2732 citations via forward citation search and amended them with 315 citations that had been excluded in review step 1 solely because they did not constitute a methodological development. Next to the exclusion of 1517 duplicates, we additionally automatically excluded 203 citations that had been excluded in review step 1 because either the title was out of scope, the study showed no gravity model, or was not in the healthcare field. We further excluded the 43 studies that had been included in step 1, since they were not application studies. Upon title screening, an additional 287 studies were excluded. Upon abstract and full text-screening, we excluded 688 studies that were lacking either a gravity model or a healthcare focus. Finally, 309 studies were included in review step 2. These 309 studies contained a total of 346 individual measures of spatial access to healthcare (Additional file 2).

### Review step 1: methodological developments

Starting from the base gravity model, two major conceptual methodological developments were proposed: The Two-Step Floating Catchment Area (2SFCA) method [11], and the Kernel Density (KD) method [30]. From 2004 onward, methodological developments almost exclusively built on the 2SFCA method and proposed developments within this family of methods, thus representing a second generation of gravity model-type methodological developments. First-generation developments (= Gravity Model Developments) and second-generation developments (= 2SFCA Developments) are discussed separately.

#### 1st Generation developments

Two major conceptual developments of the base gravity model were put forward. The 2SFCA and the KD method are conceptual developments in that 2SFCA employs predefined catchment areas that are floated over population/provider locations instead of considering all populations/providers; and KD overlays a provider and a population density layer. Mathematically however, both methods are simply special forms of the base gravity model. In addition to these conceptual developments, a small number (N = 2) of methodological papers [7, 31] proposed adaptations to the base gravity model while staying conceptually similar to it. Key characteristics of these adaptation studies are presented in Additional file 1: Appendix B.

##### Two-step floating catchment area method

*Advancement*: Floating catchment areas for determining access.

*Method*: Luo and Wang [11] introduced the Two-Step Floating Catchment Area (2SFCA) method. In the first step, all population locations within a predefined catchment area around each provider location are searched. The provider capacity is divided by the total population within the catchment area to create location-specific provider-to-population ratios. In the second step, all provider locations within the same predefined catchment area around each population location are searched. The provider-to-population ratios of all provider locations within the catchment area are summed up to build the spatial access measure. Technically speaking, the 2SFCA is a special case of the gravity model proposed by Joseph and Bantock [10] using a binary distance decay function. The function takes on a value of 1 if the provider/population point lies within the predefined catchment area (complete access assumed), and a value of 0 if it does not (no access assumed). While the base gravity model considers all providers/populations within the system, the 2SFCA method introduces the notion that beyond a certain threshold, providers are not relevant to the population anymore. Hence those providers are deemed not accessible. The reduction of providers/populations considered to only those within a catchment area implies a reduction of methodological and computational complexity. This in turn allows for more complexity and precision in the distance computation. Instead of the Euclidean distance, Luo & Wang use the estimated shortest path travel time along a road network. Interpretation of the 2SFCA access measure is in terms of providers per population, making the measure easily understandable. Adding up all 2SFCA values in the system results in the total number of providers. However, the 2SFCA measure also has drawbacks. Namely, the catchment area delineation is arbitrary. Within the catchment area, all providers are treated as equally accessible. The binary distance decay implies sharp edges where providers that are very close to each other can be treated as very differently accessible.

*Purpose*: Approximating reality.

*Methodological complexity*: Introducing catchment areas and considering only those locations within a catchment area implies a reduction of methodological complexity.

*Data requirements*: No additional data requirements.

##### Kernel density method

*Advancement*: Overlying provider and population density layers.

*Method*: Guagliardo et al. [8] introduced the Kernel Density (KD) method, which overlays provider and population density layers to compute the spatial access measure. Conceptually, this measure is thus closer to a provider-to-population ratio than the *base gravity model* or the 2SFCA. The KD method employs the Kernel Density function to create a density layer of providers out of the (in reality) discretely allocated provider points. This can be understood as a smoothing of provider points over a raster grid. Thus, the reach of providers, and hence the distance decay, is considered via the Kernel Density function. Similarly, a population density layer is created, smoothing out population data to mimic the real population distribution. Overlaying those layers, i.e., dividing the provider by the population layer results in a provider-to-population ratio for the grid cells. In the final access index, the provider-to-population ratios of all grid cells within the target area are averaged. Thus, the KD spatial access measure represents a localized provider-to-population ratio. The methodological simplicity and interpretability are clear advantages of the KD method. One downside of the KD method is that for creating the density layers, it requires somewhat arbitrarily choosing a cone radius. Another one is that the KD method does not integrate real travel behavior as travel friction is only considered in terms of Euclidean distances. However, compared to the base gravity model and the 2SFCA method, the KD method has the clear advantage of avoiding the circularity issue of other gravity models. Provider availability in Joseph and Bantock [10] and Luo and Wang [11] are adjusted according to the potential demand (i.e., size) of the surrounding population. One might argue, that for the estimation of this potential demand of the surrounding population, one would have to adjust for the potential availability of the providers surrounding those population points. Taking this line of argument further, it becomes circular. By overlaying density layers, the KD method does not suffer from this circularity issue.

*Purpose*: Approximating reality.

*Methodological complexity*: Introducing provider and population density layers implies a reduction of methodological complexity as it is conceptually close to a standard provider-to-population ratio.

*Data requirements*: No additional data requirements.

#### 2nd Generation developments (2SFCA family)

There was a plethora (N = 39) of methodological developments within the 2SFCA family. We classified them into novelty categories for which we present intuition, first study introducing the type of development, following adaptations within the category, purpose, methodological complexity, and data requirements. Additionally, all individual studies presenting a methodological development within the 2SFCA method family are given in Additional file 1: Appendix B.

##### Distance decay within catchment area

*Advancement:* Introducing a distance decay function within the catchment area.

*Intuition:* A major drawback of the 2SFCA method by Luo and Wang [11] is the binary distance decay function treating all providers within the catchment area as completely accessible and all providers outside the catchment area as not accessible. Realistically, farther away providers are less likely to be visited. To mitigate this problem and to incorporate the intuition that within a catchment area, not all providers are equally accessible, distance decay within the catchment area was introduced.

*First study:* Luo and Qi [12] built on the 2SFCA method and introduced distance decay within the catchment area. This was done to depict travel impedance more realistically. Specifically, the catchment area was split into several catchment radii, with each section being assigned a different weight. Thus, population points distant from the provider are weighted less and thus contribute less to the potential demand. Provider points distant from the population location equally are weighted less and hence contribute less to the spatial access measure. As a discrete average weight (computed by a Gaussian function) is assigned to all points lying within a sub-radius, this type of distance decay is considered a discrete stepwise distance decay.

*Development within category:* Several studies have suggested different functional forms of the decay function. This includes continuous functions (e.g., power function, Gaussian function) and a combination of a stepwise and a continuous function. Schuurman et al. [32] for example suggested no distance decay for an initial catchment radius, linear decay for the next catchment radius, and full decay (= no access) after the third threshold. Jin et al. [33] further introduced a decay function that is dependent on the provider specialization degree (i.e., they assume slower distance decay for more specialized providers). Tao et al. [34] assumed that the distance decay function varies by rurality (i.e., they assume slower distance decay in rural areas). The developments concerning distance decay have been synthesized by Wang [21], who suggested a generalized 2SFCA. Instead of specifying a particular distance decay function, the generalized 2SFCA introduced a general decay function, leaving it up to the researcher to decide which empirical functional form fits best their context and assumptions about distance decay. The generalized 2SFCA consolidated previous measures, formalizing the general principle used by other authors.

*Purpose:* Approximating reality.

*Methodological complexity:* Introducing a (stepwise, continuous, or hybrid) distance decay function within the catchment area moderately increases modeling complexity. In addition to determining for each provider-population pair whether they are within the catchment area threshold distance, a distance decay function must be applied for each provider-population pair within the catchment area.

*Data requirements:* No additional data requirements.

##### Variable catchment area sizes

*Advancement:* Variable catchment size definition.

*Intuition:* Luo and Wang’s [11] 2SFCA formulation assumed fixed and uniform catchment area sizes for all provider and population locations. However, urban and rural populations might have a differing willingness to travel due to the presence of nearby providers. In other words, when providers are far away, patients will travel farther to get access to healthcare; when many providers are nearby, the willingness to travel will be lower.

*First study:* Luo and Whippo [13] introduced variable catchment sizes to the 2SFCA method (= V2SFCA). This was done to reflect differences in surrounding provider density leading to differences in accepted initial travel time. They proposed to dynamically determine catchment sizes by increasing a catchment size until a predefined provider-to-population threshold is met. This specification cannot result in population locations with zero spatial access. This fully variable catchment size depends both on the provider capacity and the population density and results in unique catchment sizes for each provider/population location. More generally speaking, in the variable 2SFCA the catchment threshold is a function of certain characteristics of the provider/population location.

*Development within category*: Reflecting differences in catchment thresholds for urban and rural populations has been conducted in different ways. While Luo and Whippo [13] defined catchment sizes dependent on a base provider-to-population threshold, McGrail and Humphreys [35] chose to define discrete catchment sizes. They suggested to assign one of five catchment area sizes to a provider/population location based on the rurality classification of that location. Variable catchment sizes have not only been implemented to depict urban and rural differences, but also to account for varying reach depending on provider specialization degrees. Such approaches reflect that more specialized or larger providers serve a larger population. Kim et al. [36] for example used the number of physicians per hospital as determinant for the catchment size, while Tao et al. [37] defined the catchment area size as dependent on the level of hospital specialization.

*Purpose:* Approximating reality.

*Methodological complexity:* Introducing variable catchment sizes only slightly increases modeling complexity. The criterion for determining whether a provider-population pair is within a catchment area shifts from a single condition (e.g., less than 30 min driving time) to combined conditions (e.g., less than 30 min driving time for population in urban area and less than 60 min driving time for population in rural area).

*Data requirements:* Additional data may be required in some cases, e.g., a rurality classification for all provider and population locations. Additional data is not required when catchment sizes are determined conditional on a base provider-to-population threshold.

##### Outcome unit modification

*Advancement:* Outcome unit modification to interpretation in relative terms.

*Intuition:* Accounting for distance decay within a catchment area (like in the generalized 2SFCA) requires the definition of a distance impedance parameter. Since empirical verification of distance disutility is difficult, the choice of the impedance parameter is somewhat arbitrary. Yet, different values of the distance impedance parameter can vastly alter the magnitude of the spatial access measure. To account for this uncertainty, an outcome unit modification was proposed.

*First study:* Wan, Zhan et al. [38] proposed to compute a spatial access ratio (SPAR) from the 2SFCA measure. The SPAR is defined as the ratio between a population location’s spatial access measure and the mean spatial access measure of all population locations. SPAR thus represents a dimensionless relative outcome measure rather than an absolute provider-to-population-like measure. SPAR is stable to different distance impedance parameter specifications and useful for mapping when no absolute outcome values are needed. This development comes at no cost in terms of data requirements or computation.

*Development within category:* No further studies suggested an outcome unit modification.

*Purpose:* Correcting methodology.

*Methodological complexity:* Introducing an outcome unit modification does not increase modeling complexity. An additional step is required to compute the Spatial Access Ratio. However, it requires simply dividing the population location’s access index by the mean access measure of all population locations, thus not noticeably adding complexity.

*Data requirements:* No additional data requirements.

##### Provider competition

*Advancement:* Correcting demand overestimation by introducing a provider competition-based selection weight.

*Intuition:* In the first step of 2SFCA, all population locations within a provider’s catchment area are considered to contribute to the potential demand at that provider location. Thus, population points may be counted fully as contributing to the potential demand at several provider locations, implying an overestimation of demand. However, individuals do not visit all accessible providers, but rather choose one provider. To correct the implicit demand overestimation, a provider competition scheme was suggested. This scheme assigns populations based on a choice probability, reflecting that populations face several provider options.

*First study:* Wan, Zou, and Sternberg [39] proposed the three-step floating catchment area method (3SFCA) which computes a distance impedance-based selection weight for each provider-population pair in an additional first step. This approach considers all surrounding providers (and distances to them) a population can choose from in its catchment area. As such, the potential demand more accurately depicts the actual demand on a provider. The closer a provider is to a population location, the more likely the population will be to visit the provider; hence the selection weight assigns a larger share of the population to closer providers. The additional step is computed using a Huff-model based selection weight.

*Development within category:* While the initial 3SFCA proposed by Wan, Zou, and Sternberg [39] considered only distance impedance as determinant of the selection probability, other developments incorporated additional factors into the provider competition scheme. Luo [40] amended the selection weight computed in the first step by including provider capacity in addition to the distance impedance function. Other functions for the computation of the selection probability have also been proposed, incorporating a plethora of factors determining the selection weight [e.g., 41–43]. Notably, Paez, Higgins, and Vivona [44] showed that standard 2SFCA methods not only overestimate demand, but also overestimate supply as the provider capacity computed in the provider-to-population ratio may be counted to the final access score at several population locations. Thus, instead of computing a selection probability to be applied in the provider-to-population ratio, Paez et al. [44] fully proportionally allocated population and providers to account for potential demand and supply inflation. This is done by standardizing the distance decay weights within the distance decay function in each catchment area to 1. Hence the total level of demand and supply within the system are preserved in this measure.

*Purpose:* Correcting methodology.

*Methodological complexity:* Introducing a selection weight heavily increases modeling complexity. An additional first step is required to compute the selection weight. In addition to computing a provider-to-population ratio at each provider location considering all surrounding population locations, a selection weight for each provider-population pair considering all surrounding provider locations must be computed. This almost doubles computational intensity.

*Data requirements:* No additional data requirements.

##### Local and global distance decay

*Advancement:* Correcting for sub-optimally configured healthcare system by modeling local and global distance decay.

*Intuition:* Delamater [45] showed that the standard configuration of 2SFCA methods only considers relative distances within a catchment area and ignores absolute distances. That is, only the relative distance differences among providers within the population’s catchment area impact the final access score, not the absolute distance differences across catchment areas. In a simple example, population locations with only one provider within the catchment area would receive the exact same spatial access score, irrespective of whether the provider is close by or far away. This implicitly assumes optimally configured healthcare systems, where population locations do not face different absolute distances to providers. Arguably, even if two population locations have the same provider availability within their catchment area, we might consider a population location from which those providers are farther away to have poorer accessibility.

*First study:* Delamater [45] put forward a methodological development to correct for sub-optimally configured healthcare systems as they are found in reality. By introducing an additional distance decay function in the first step, the method produces specific pairwise supply ratios rather than single supply ratios per provider and thus accounts for both relative and absolute distances. Conceptually, this can be understood as having a local distance decay function (= relative distances) and a global distance decay function (= absolute distances).

*Development within category:* Bauer and Groneberg [46] further advanced this method by using two different functional forms for the local and the global distance decay function. Assumptions about the nature of the relative distance decay within the catchment area are formalized in the local decay function, while assumptions about the role of absolute distance are captured in the global distance decay function. This allows to differentially conceptualize the role of relative and absolute distances.

*Purpose:* Correcting methodology.

*Methodological complexity:* Introducing an additional distance decay function slightly increases modeling complexity, more so, if different functional forms are used for the local and global distance decay functions.

*Data requirements:* No additional data requirements.

##### Subgroup-specific access

*Advancement*: Subgroup-specific access measure for selective population-provider pairing.

*Intuition:* Factors other than spatial impedance and provider capacity have an impact on whether an individual can access a provider, i.e., not all providers are available to all populations equally. This might be due to e.g., language restrictions, insurance plans, or referral systems. In turn, subgroups of individuals compete for a subgroup of providers, both of which may exhibit spatial variation. Standard 2SFCA methods, however, assume equal non-spatial access for all populations to all providers.

*First study:* Wang [47] proposed an additional step after computing the access measure for quantifying group-specific access of Chinese immigrants to ethnic Chinese physicians. This was done by considering the relative abundance of ethnic Chinese physicians as well as the share of Chinese immigrants within the catchment area of a population location. Thus, demand competition of a population subgroup for a provider subgroup is reflected.

*Development within category:* Other context-specific methodological developments also reflected that not all providers are available to all populations equally. Xiao et al. [48] integrate referrals within a hierarchical healthcare system where higher specialized providers can only be accessed upon referral. Shao and Luo [49] incorporated group-specific healthcare access resulting from different health insurance plans. In their method, a provider’s resources are only counted toward the accepted insurance plan of a population subscribed to that same plan. The resulting measure yields access scores by insurance plan.

*Purpose:* Fitting context.

*Methodological complexity:* Introducing a subgroup-specific access index heavily increases modeling complexity. An additional third step is required to compute the subgroup-specific index. In addition to computing the standard 2SFCA measure, a weight considering all surrounding subgroup-specific provider and population locations must be computed. This almost doubles computational intensity.

*Data requirements:* Additional data are required for information on subgroup-specific provider and population shares at each location.

##### Multiple transportation modes

*Advancement:* Multiple transportation modes.

*Intuition:* Standard 2SFCA methods considered one transportation mode only—typically, travel time along a road network by private vehicle was used to model distance impedance. This is a simplified assumption. For one, traveling by public transport or by foot implies different travel times and thus accessibility. For another, population locations differ in terms of mobility, i.e., the share of individuals using each mode of transport.

*First study:* Mao and Nekorchuk [14] suggested a multi-mode 2SFCA which incorporates multiple transportation modes that individuals might use to reach a provider. The method captures several transport mode options as well as varying mobility by population location. Specifically, transport-mode specific catchment sizes are defined (e.g., 30-min travel time by private vehicle and 60-min travel time by public transit). In the first step, for each transport mode, only population points within the mode-specific catchment are considered, and only the assumed mode-using share of the population is counted to build a mode-specific provider-to-population ratio. These mode-specific ratios are summed up to form the full provider-to-population ratio. The same approach is used for the second step, additionally weighting the mode-specific reachable provider ratios by the corresponding mode-using share of the population at the location. As such, this measure can also account for competition among populations with differing mobility.

*Development within category:* Producing a more individualized measure, Langford, Higgs, and Fry [50] suggested computing separate access measures for each transport mode. When computing provider-to-population ratios in the first step, all transportation modes are considered (just like in the Mao & Nekorchuk [14] method). In the second step, only the mode-specific reachable provider ratios are summed up. This allows to incorporate demand competition for providers, while producing travel mode-specific scores. Zhou et al. [51] incorporated different travel mode choice probabilities for each provider-to-population pair instead of simply using different fractions of mode users by transport type per population location, thus producing a more detailed measure.

*Purpose:* Approximating reality.

*Methodological complexity:* Introducing multiple transportation modes heavily increases modeling complexity. For each provider-population pair, travel distance/time must be computed for at least two modes of transportation. Additionally, demand intensity is weighted by the mode-specific user shares at each population location. This at least doubles computational intensity.

*Data requirements:* Additional data are required for information on travel distance/time by transport mode as well as data on mode-specific user shares for each population location.

##### Time-dependent access

*Advancement:* Spatio-temporal access measure using time-varying parameters.

*Intuition:* Previous 2SFCA methods were static in the sense that no temporal variability of the input parameters was considered. Individuals might face greatly varying spatial access depending on the time of day or year due to e.g., traffic congestion, opening hours, or road conditions. Spatio-temporal access measures explicitly model temporal variability, producing a range of access measures rather than relying on one static measure which reflects spatial access only at a specific time point.

*First study:* Ma et al. [52] introduced a temporal dimension in their 2SFCA measure by incorporating time-varying travel times. Using real-time traffic data at different time points within the same day, they captured varying traffic congestion. Thus, the travel impedance becomes a time-dependent parameter in this method. While previous 2SFCA measures frequently assumed average travel speeds for road segments to compute travel times, this method relies on routing algorithms using empirical travel speeds. Importantly, this approach does not produce one combined static access measure. Instead, it gives a range of access measures that depict spatial access at different time points.

*Development within category:* Instead of looking at within-day variability, Song et al. [53] incorporated seasonal differences in average travel times due to varying precipitation levels. Further developments incorporated time-varying demand-size [54] and time-varying supply size [55]. The former aims to reflect variable demand intensity due to commuting behavior by using mobile phone GPS data [54]. The latter reflects variable provider availability throughout the day by incorporating provider opening hours [55].

*Purpose:* Approximating reality.

*Methodological complexity:* Methodological complexity is not affected by this development as the basic configurations of the 2SFCA method remain unchanged. However, as this development requires generating a range of access measures with different parameter inputs computational intensity will be heavily affected.

*Data requirements:* Additional data are required for information on time-varying travel times, population size, or provider opening hours (Table 1).

**Table 1:** Methodological developments in the 2SFCA family

Category	Advancement	Studies	Formula^†^	Purpose	Complexity	Data requirements
Distance Decay within Catchment Area	Introduction of distance decay within the catchment area	First study: • *E2SFCA* Luo & Qi, 2009 [12] Development within category: • McGrail & Humphreys, 2009 [72] • Dai, 2010 [67] • Schuurman et al., 2010 [32] • Plachkinova et al., 2018 [73] • Jin et al., 2019 [33] • Tao et al., 2020 [34]	${R}_{j}=\frac{{S}_{j}}{{\sum }_{r}{\sum }_{i \in \{{d}_{ij}\le {d}_{r}\}}{P}_{i}*{W}_{r}}$ ${A}_{i}^{*}= {\sum }_{r}{\sum }_{j \in \{{d}_{ij}\le {d}_{r}\}}{R}_{j}*{W}_{r}$	Approximating Reality	Moderate increase of modeling complexity	No additional data required
Variable Catchment Area Sizes	Variable catchment size definition	First study: • *V2SFCA* Luo & Whippo, 2012 [13] Development within category: • McGrail & Humphreys, 2014 [35] • Jamtsho et al., 2015 [68] • Ni et al., 2015 [74] • Kim et al., 2018 [36] • Tao et al., 2018 [37] • Bozorgi et al., 2021 [75]	${R}_{j}=\frac{{S}_{j}}{{\sum }_{i \in \{{d}_{ij}\le {d}_{x}({P}_{i})\}}{P}_{i}*f({d}_{ij},\beta )}$ ${A}_{i}^{*}={\sum }_{j \in \{{d}_{ij}\le {d}_{x}({R}_{j})\}}{R}_{j}*f({d}_{ij},\beta )$	Approximating Reality	Slight increase of modeling complexity	May require additional data
Outcome Unit Modification	Outcome unit modification to relative terms	First study: • *SPAR* Wan, Zhan et al., 2012 [38] Development within category: –	${R}_{j}=\frac{{S}_{j}}{{\sum }_{i \in \{{d}_{ij}\le {d}_{0}\}}{P}_{i}*f({d}_{ij},\beta )}$ ${A}_{i}^{*}= {\sum }_{j \in \{{d}_{ij}\le {d}_{0}\}}{R}_{j}*f({d}_{ij},\beta )$ ${A}_{i}^{SPAR}= \frac{{A}_{i}^{*}}{{A}_{\varnothing }}$	Correcting Methodology	No increase of modeling complexity	No additional data required
Provider Competition	Correcting demand overestimation by introducing a provider competition-based selection weight	First study: • *3SFCA* Wan, Zou et al., 2012 [39] Development within category: • Luo, 2014 [40] • Tang et al., 2017 [43] • Paez et al., 2019 [44] • Matthews et al., 2020 [76] • Jang, 2021 [41] • Shen et al., 2021 [42]	${Prob}_{ij}= \frac{f\left({d}_{ij}, \beta \right)}{{\sum }_{i \in \{{d}_{ij}\le {d}_{0}\}}f\left({d}_{ij}, \beta \right)}$ ${R}_{j}=\frac{{S}_{j}}{{\sum }_{i \in \{{d}_{ij}\le {d}_{0}\}}{P}_{i}*f\left({d}_{ij},\beta \right)*{Prob}_{ij}}$ ${A}_{i}^{*}= {\sum }_{j \in \{{d}_{ij}\le {d}_{0}\}}{R}_{j}*f({d}_{ij},\beta )$	Correcting Methodology	High increase of modeling complexity	No additional data required
Local & Global Distance Decay	Correcting for sub-optimally configured healthcare system by modeling local and global distance decay	First study: • *M2SFCA* Delamater, 2013 [45] Development within category: • Bauer & Groneberg, 2016 [46]	${R}_{j}=\frac{{S}_{j}*f({d}_{ij},\beta )}{{\sum }_{i \in \{{d}_{ij}\le {d}_{0}\}}{P}_{i}*f({d}_{ij},\beta )}$ ${A}_{i}^{*}= {\sum }_{j \in \{{d}_{ij}\le {d}_{0}\}}{R}_{j}*f({d}_{ij},\beta )$	Correcting Methodology	Slight increase of modeling complexity	No additional data required
Subgroup-Specific Access	Subgroup-specific access measure for selective population-provider pairing	First study: • *Subgroup-specific 2SFCA* Wang, 2007 [47] Development within category: • Xiao et al., 2021 [48] • Yang et al., 2021 [69] • Shao & Luo, 2022 [49]	${R}_{j}=\frac{{S}_{j}}{{\sum }_{i \in \{{d}_{ij}\le {d}_{0}\}}{P}_{i}*f({d}_{ij},\beta )}$ ${A}_{i}^{*}= {\sum }_{j \in \{{d}_{ij}\le {d}_{0}\}}{R}_{j}*f({d}_{ij},\beta )$ ${AG}_{i}^{*}={A}_{i}^{*}$ $*\frac{{\sum }_{j \in \{{d}_{ij}\le {d}_{0}\}}{SG}_{j}*f({d}_{ij},\beta )}{{\sum }_{j \in \{{d}_{ij}\le {d}_{0}\}}{S}_{j}*f({d}_{ij},\beta )}/\frac{{\sum }_{i \in \{{d}_{ij}\le {d}_{0}\}}{PG}_{i}*f({d}_{ij},\beta )}{{\sum }_{i \in \{{d}_{ij}\le {d}_{0}\}}{P}_{i}*f({d}_{ij},\beta )}$	Fitting Context	High increase of modeling complexity	Additional data on subgroup-specific provider and population shares required
Multiple Transportation Modes	Multiple transportation modes	First study: • *MM-2SFCA* Mao & Nekorchuk, 2013 [14] Development within category: • Polzin et al., 2014 [77] • Langford et al., 2016 [50] • Ni et al., 2019 [78] • Tao & Cheng, 2019 [79] • Zhou et al., 2020 [51] • Xing & Ng, 2022 [80]	$${R}_{j}=\frac{{S}_{j}}{{\sum }_{n}{\sum }_{\begin{array}{c}i \in \{{d}_{ij\left({M}_{n}\right)}\\ \le {d}_{0{(M}_{n})}\}\end{array}}{P}_{i({M}_{n})}*f({d}_{ij},\beta )}$$ $${A}_{i}^{*}={\sum }_{n}\frac{{P}_{i\left({M}_{n}\right)}}{{P}_{i}}{\sum }_{\begin{array}{c}j \in \{{d}_{ij\left({M}_{n}\right)}\\ \le {d}_{0{(M}_{n})}\}\end{array}}{R}_{j}*f({d}_{ij},\beta )$$	Approximating Reality	High increase of modeling complexity	Additional data on mode-specific distances and mode-specific user shares required
Time-Dependent Access	Dynamic time-varying access measure	First study: • *Spatio-Temporal 2SFCA* • Ma et al., 2018 [52] Development within category: • Song et al. 2018 [53] • Paul & Edwards, 2019 [55] • Xia et al., 2019 [54]	${R}_{jt}=\frac{{S}_{j}}{{\sum }_{i \in \{{d}_{ij({T}_{t})}\le {d}_{0}\}}{P}_{i}*f({d}_{ij({T}_{t})},\beta )}$ ${A}_{it}^{*}= {\sum }_{j \in \{{d}_{ij({T}_{t})}\le {d}_{0}\}}{R}_{j}*f({d}_{ij({T}_{t})},\beta )$	Approximating Reality	No increase of modeling complexity	Additional data on time-varying travel time, provider supply or population size required

${S}_{j}$: healthcare capacity (= supply) at location $j, {P}_{i}$: demand intensity (= population) location $i, {d}_{ij}$: distance between population location $i$ and provider location $j, {d}_{r}$: threshold distance of catchment area sub-radius $r, {W}_{r}$: distance decay weight within catchment area sub-radius $r, {R}_{j}$: provider-to-population ratio at location $j, {A}_{i}^{*}$: access index at location $i, \beta$: distance friction parameter, $f\left(\right)$: general distance decay function, dependent on distance and distance friction, ${d}_{x}\left(\right)$: threshold distance of catchment area, dependent on characteristics of population/provider location, ${A}_{\varnothing }$: mean access index in study area, ${A}_{i}^{SPAR}$: Spatial Access Ratio at location $i, {Prob}_{ij}$: distance-impedance-based selection weight for population $i$ and provider $j$ pair, ${SG}_{j}$: subgroup-specific healthcare capacity (= supply) at location $j, {PG}_{i}$: subgroup-specific demand intensity (= population) location $i, {AG}_{i}^{*}$: subgroup-specific access index at location $i, {P}_{i\left({M}_{n}\right)}$: demand intensity (= population) location $i$ using transportation mode $n, {d}_{ij\left({M}_{n}\right)}$: distance between population location and provider location using transportation mode $n, {d}_{0\left({M}_{n}\right)}$: threshold distance of catchment area for transportation mode $n, {d}_{ij\left({T}_{t}\right)}$: distance between population location $i$ and provider location $j$ at time point $t, {R}_{jt}$: provider-to-population ratio at location $j$ at time point $t, {A}_{it}^{*}$: access index at location *i* at time point $t$

*E2SFCA* Enhanced Two-Step Floating Catchment Area, *V2SFCA* Variable Two-Step Floating Catchment Area, *SPAR* Spatial Access Ratio, *3SFCA* Three-Step Floating Catchment Area, *M2SFCA* Modified Two-Step Floating Catchment Area, *Subgroup-specific 2SFCA* Subgroup-specific Two-Step Floating Catchment Area, MM-2SFCA Multi-Mode Two-Step Floating Catchment Area

^**†**^Formula refers to the methodology proposed in the first study of each category. The formal notation of the methodological developments was harmonized to provide consistency, hence can differ from the notations given in the cited studies

### Review step 2: application studies

#### Study characteristics

**Fig. 2:**
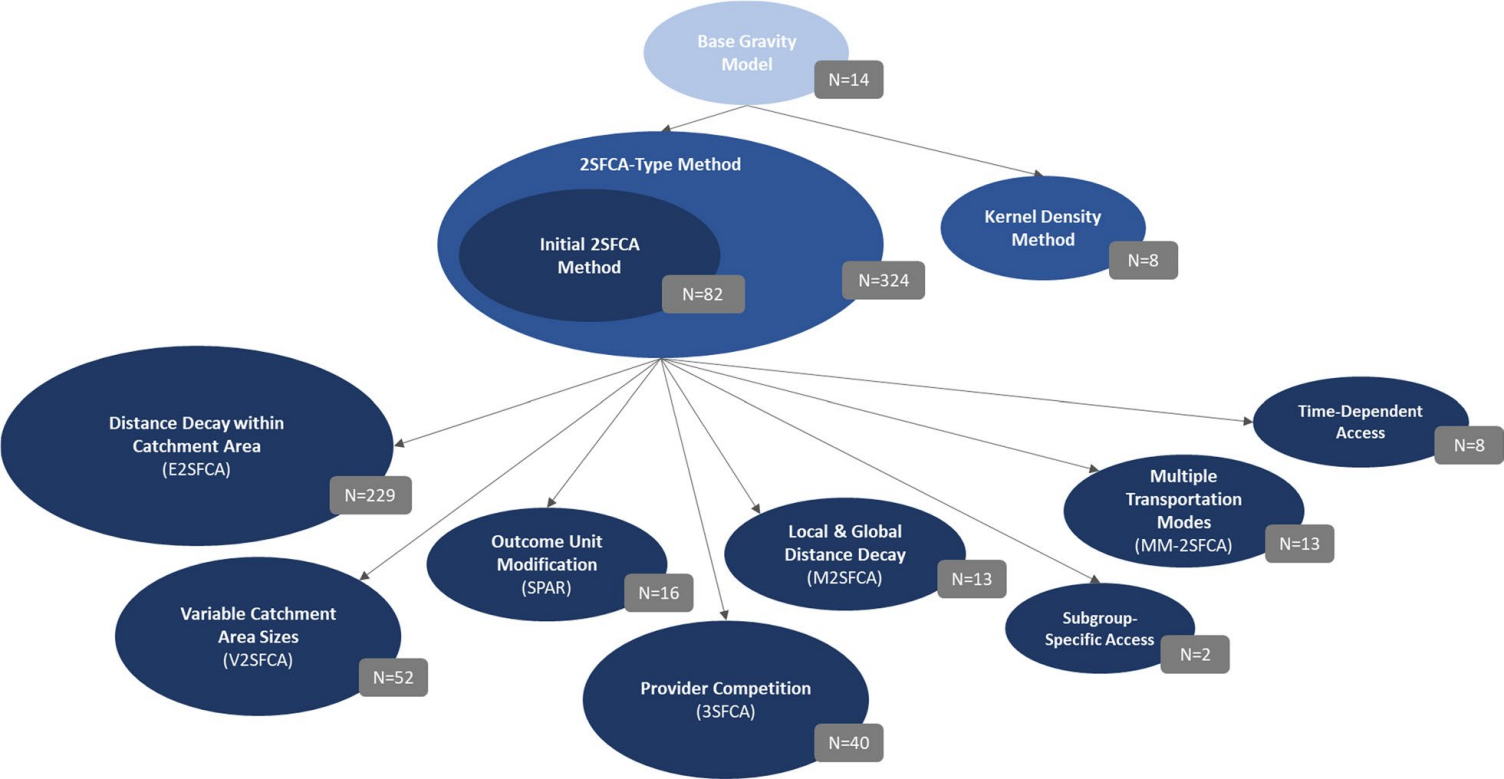
Lineage and application frequency of gravity model developments. Displayed in light blue is the base gravity model, displayed in blue are the conceptual developments derived from it (2SFCA method, Kernel Density method), displayed in dark blue are categories of methodological developments derived from the 2SFCA method. Number of access measures that applied the respective methodological development is given in gray boxes, bubble sizes are weighted by frequency of application. Each applied measure was assigned to either the base gravity model, the 2SFCA method family, or the Kernel Density method (= mutually exclusive). In addition, for all 324 2SFCA-type measures, the methodological development(s) applied were identified. One 2SFCA-type measure can implement more than one methodological development (= not mutually exclusive). Therefore, the sum of 2SFCA-type methodological developments applied exceeds the number of measures classified as 2SFCA-type. *2SFCA* Two-Step Floating Catchment Area, *E2SFCA* Enhanced Two-Step Floating Catchment Area, *V2SFCA* Variable Two-Step Floating Catchment Area, *SPAR* Spatial Access Ratio, *3SFCA* Three-Step Floating Catchment Area, *M2SFCA* Modified Two-Step Floating Catchment Area, *MM-2SFCA* Multi-Mode Two-Step Floating Catchment Area

The 309 application studies entailed a total of 346 individual measures that constituted applications of the earlier identified methodological developments. Since the application of specific methods is of interest here, the following numbers refer to individual measures rather than studies. Frequency statistics on the characteristics of access measures are given in Table 2. An overview of the measures applied by methodological development type is given in Fig. 2. Most healthcare access measures were applied either in an Asian (N = 160; 46%) or a North American (N = 136; 39%) healthcare setting, with USA (N = 109), China (N = 91), and Canada (N = 27) being the most frequently investigated countries. In 45% of the application cases (N = 157), spatial access to providers within the primary care sector was investigated, 39% of the measures (N = 135) were applied to secondary care providers and 16% (N = 54) to both primary and secondary care providers. Provider capacity was measured in a variety of ways, including physician head counts (e.g., [56]), physician full-time equivalents (e.g., [15]), number of hospital beds (e.g., [57]), number of sites (e.g., [29]), or number of services (e.g., [58]) available. Most spatial access measures (N = 186; 54%) were applied to a geographic scope covering both urban and rural regions, while 41% (N = 142) were applied to an exclusively urban, and 5% (N = 18) to an exclusively rural context. To operationalize travel friction in the gravity model, most applications (72%; N = 249) used the estimated shortest travel time along a road network between population and provider location. Another 16% (N = 56) of the measures incorporated travel friction by means of estimated shortest travel distance, and 12% (N = 41) used Euclidean distance. Almost all spatial access measures (98%, N = 338) were computed within the scope of the respective studies. However, a small share of studies relied on secondary data (2%; N = 8), using access indices that had been previously computed by other researchers.^1^ There was a range of purposes that the healthcare access measures were applied for: 55% (N = 189) of the measures served the purpose of describing the spatial access in a particular healthcare system. In 19% of the cases (N = 67), the spatial access measure was used as a covariate to explain health outcomes (e.g., [59]). Another 15% of the measures (N = 51) were applied in a methodologically exploratory way; that is, investigating model properties by applying different parameters (e.g., [60]). Lastly, 11% (N = 39) of the measures were applied to conduct a comparison of methods, either comparing gravity model indicators to non-gravity type accessibility or availability measure (e.g., [61]), or comparing several different gravity model methodologies (e.g., [62]).

**Table 2:** Characteristics of measures applying gravity model developments

Variables	Total	Base gravity model	2SFCA method family	Kernel density method
Case numbers	346	14	324	8
*Study area*				
Africa	4 (1%)	0 (0%)	4 (1%)	0 (0%)
Asia	160 (46%)	10 (71%)	148 (46%)	2 (25%)
Europe	36 (10%)	0 (0%)	36 (11%)	0 (0%)
North America	136 (39%)	3 (21%)	128 (40%)	5 (63%)
South America	10 (3%)	1 (7%)	8 (2%)	1 (13%)
*Care sector*				
Primary	157 (45%)	1 (7%)	153 (47%)	3 (38%)
Secondary	135 (39%)	8 (57%)	124 (38%)	3 (38%)
Primary and secondary	54 (16%)	5 (36%)	47 (15%)	2 (25%)
*Study area region type*				
Urban	142 (41%)	10 (71%)	127 (39%)	5 (63%)
Rural	18 (5%)	2 (14%)	16 (5%)	0 (0%)
Urban and rural	186 (54%)	2 (14%)	181 (56%)	3 (38%)
*Distance type*				
Travel time	249 (72%)	9 (64%)	238 (73%)	2 (25%)
Travel distance	56 (16%)	2 (14%)	54 (17%)	0 (0%)
Euclidean distance	41 (12%)	3 (21%)	32 (10%)	6 (75%)
*Indicator computation*				
Primary computation	338 (98%)	14 (100%)	316 (98%)	8 (100%)
Secondary use	8 (2%)	0 (0%)	8 (2%)	0 (0%)
*Application case*				
Descriptive	189 (55%)	8 (57%)	178 (55%)	3 (38%)
Covariate	67 (19%)	2 (14%)	63 (19%)	2 (25%)
Exploratory	51 (15%)	2 (14%)	48 (15%)	1 (13%)
Comparison	39 (11%)	2 (14%)	35 (11%)	2 (25%)

*2SFCA* two-step floating catchment area

#### Application frequency: 1st generation developments

Applications of gravity models for spatial healthcare access measurement were almost exclusively restricted to methods from within the 2SFCA family (94%, N = 324). Only few measures applied a base gravity model or the KD method: 4% (N = 14) of the applications represented measures derived from the base gravity model method and 2% (N = 8) applied the KD method to measure potential spatial access to healthcare.^2^

#### Application frequency: 2nd generation developments (2SFCA family)

Out of the 324 measures implementing a 2SFCA-type methodology, 25% (N = 82) computed the measure exactly like Luo and Wang [11] had first suggested it. That is, no distance decay within the catchment area, fixed catchment sizes across the study context, no outcome unit modification, and no additional assumptions or corrections in terms of provider competition, subgroup-specific access, multiple transportation modes, or time-dependent access were made. For the remaining 242 2SFCA-type measures that implemented further methodological developments, distance decay within the catchment area was the most frequently applied (71%, N = 229), followed by implementations of variable catchment areas (16%, N = 52), and incorporation of provider competition (12%, N = 40). Modification of the outcome unit (5%, N = 16) and considering local and global distance decay functions (4%, N = 13), subgroup-specific access measures (< 1%, N = 2), multiple transportation modes (4%, N = 13), and time-dependent access (2.5%, N = 8) were implemented to a small degree only. The application frequency of 2SFCA-type methodological developments is given in Table 3.

**Table 3:** Application frequency of 2SFCA-type methodological developments

Methodological development	N of 2SFCA-type measures applied this methodological development	% of 2SFCA-type measures applied this methodological development
2SFCA-type measures applied (Total)	324	100
Initial 2SFCA (No methodological development)	82	25
Distance decay within catchment area	229	71
Variable catchment area sizes	52	16
Outcome unit modification	16	5
Provider competition	40	12
Local and global distance decay	13	4
Subgroup-specific access	2	1
Multiple transportation modes	13	4
Time-dependent access	8	2

For all 324 2SFCA-type measures, the methodological development(s) applied were identified. One 2SFCA-type measure can implement more than one methodological development (= not mutually exclusive). Therefore, the sum of 2SFCA-type methodological developments applied exceeds the number of measures classified as 2SFCA-type

*2SFCA* two-step floating catchment area

##### Distance decay

With more than half of all the 2SFCA applications accounting for distance decay within the catchment area, this methodological development has caught on widely in research practice. The stepwise discrete approach as proposed by Luo and Qi [12] was implemented in 96 access measures. In it, a fixed distance decay weight is assigned to each location according to the catchment area sub-radius it is in. Another 105 measures applied a continuous distance decay function within the catchment area. Particularly common examples of functional forms were Gaussian functions and power functions. The remaining 28 access measures implemented a hybrid form in which both a fixed distance decay weight and a continuous distance decay function were used within the catchment area. Such hybrid forms usually assume zero decay within a certain distance threshold and apply a continuous decay function thereafter.

##### Catchment area

Of the 52 measures applying variable catchment area definitions, 31 operationalized catchment areas to depend on a categorical variable. Typical examples would be catchment sizes depending on a categorical urban–rural classification or depending on hospital type. The other 21 measures defined catchment area sizes in a continuous fashion, e.g., dependent on the number of doctors at a provider location (larger healthcare institutions have a wider reach) or dependent on fulfilment of a minimally required provider-to-population ratio (higher distance tolerance in low-density areas).

##### Outcome unit

A small fraction of the 2SFCA type methods applied a modification of the outcome unit, i.e., dividing the spatial access measure at a population location by the mean spatial access measure. That is, these 16 studies computed a relative access measure e.g., for comparing regions.

##### Provider competition

Out of the measures using a selection weight to consider provider competition, 34 measures incorporated a Huff-model based selection probability in an additional first step akin to the 3SFCA proposed by Wan, Zou et al. [39]. In that additional first step, a selection probability for each provider-population pairing is computed using the surrounding providers at a particular population location. Computation of these selection probabilities is either based on relative distances to surrounding providers only [e.g., 57], i.e., closer providers are assumed to more likely be visited. Or both distance and capacity are considered [15], i.e., providers with more resources additionally are assumed to be visited more. The remaining 6 measures followed the methodology suggested by Paez et al. [44] in fully allocating supply and demand to account for provider competition. That is, each demand unit (e.g., one inhabitant) and each supply unit (e.g., one pediatrician) are considered only once as contributing to the potential demand on a provider, and to the potential availability for a population, respectively.

##### Local and global distance decay 

Using both local and global distance decay functions to model both the relative and absolute distances to providers was rare. Of those measures implementing it, 7 measures accounted for relative and absolute distances like Delamater [45] by adding an additional distance decay term with the same functional form in the first step of the method. The local and global distance friction are assumed to show the same decay properties. The other 6 measures followed the methodology put forward by Bauer and Groneberg [46] who introduced separate functions for the local and the global distance decay.

##### Subgroup-specific access

Almost no measures implemented a methodology with a subgroup-specific access measure. Two studies used an additional third step to compute a subgroup specific access to healthcare providers with matching language or ethnicity. No measures were found to provide e.g., insurance-specific access indices or access indices considering referral within a hierarchical healthcare system.

##### Multiple transportation modes

Of those measures incorporating multiple transportation modes, 9 measures produced an integrated spatial access indicator for all transportation modes. That is, the final indicator in those studies was a weighted measure which not only includes travel times differing by transport mode, but also varying shares of mode-specific users per population location. The remaining 4 measures produced separate spatial access indices for each transportation mode. Of those, 3 assumed no differences in mobility across population locations and applied the same methodology with the same parameters once for each transportation mode. The other 1 study computed mode-specific access measures which considered demand competition arising from varying ranges by different transport-mode users, i.e., which incorporated varying shares of mode-specific users for population locations.

##### Time-dependent access

Time-dependent access was incorporated to a relatively small degree so far. However, this is the youngest category of methodological developments being proposed for the first time in 2018. Among the studies applying a time-dependent approach, there was wide variation in how time-dependency was accounted for. Some measures (N = 3) employed time-dependent travel times using real-time traffic data. Other measures used time-dependent demand [63], time-dependent supply [64], or a combination of time-dependent travel time, demand, and supply [65, 66].

## Discussion

### Summary of key findings

In review step 1, we identified 43 studies that proposed a methodological development in the field of gravity models for measuring spatial access to healthcare. Starting from a base gravity model that integrated availability and accessibility as well as supply and demand factors, first proposed by Joseph and Bantock [10], two major conceptual developments emerged: the two-step floating catchment area (2SFCA) method [11] and the Kernel Density method [30]. While both methods are special forms of the base gravity model, these two developments are conceptually different. From 2004 onward, virtually all methodological developments were based on the 2SFCA method. These studies proposed methodological developments regarding distance decay within the catchment area, variable catchment area sizes, outcome unit modification, provider competition, local and global distance decay, subgroup-specific access, multiple transportation modes, and time-dependent access.

In review step 2, we identified 309 application studies covering a total of 346 measures. Application studies focused almost exclusively on measures from the 2SFCA family. The usage of 2SFCA type methods to describe spatial healthcare access has caught on widely in the health research practice. In comparison, the base gravity model (and its variants) as well as the KD method have found much less application in research practice. Among the studies employing an access measure from the 2SFCA family, most implement some kind of distance decay within the catchment area. Other frequently implemented methodological developments from the 2SFCA family were variable catchment area sizes, and selection probabilities to account for provider competition. However, usage of the basic 2SFCA method as it was developed by Luo and Wang [11] was still very common.

### Appraisal

#### Review step 1: methodological developments

We identified three types of purpose the methodological developments were serving: Approximating reality, fitting context, and correcting methodology.

*Approximating reality:* Many of the methodological developments stem from attempts to make the spatial access measure better reflect reality. Distance decay within the catchment area (e.g., [12, 32, 67]) better reflects real-world travel friction; variable catchment area sizes allow for integration of real-world differences in willingness to travel (e.g., [13, 35, 68]); multiple transportation modes allow for modeling real-world travel behavior (e.g., [14, 50, 51]); and time-dependent access captures real-world temporal variability in access (e.g., [64–66]). While this makes spatial access measures more realistic, it also comes at a cost. Firstly, it often means additional methodological complexity, higher computational intensity, or additional data requirements. For example, accounting for multiple transportation modes not only means additional computational power required, but also additional data requirements. Secondly, within increasing methodological complexity, also the interpretation of the final access score may become more complex. In the case of the Variable 2SFCA for example, varying distance tolerance is assumed due to longer travel times some populations must accept to reach any provider. Hence, the final access score refers to spatial access e.g., within a 30-min radius in some locations and a 60-min radius in others. Thirdly, additional assumptions mean additional uncertainty. Integrating methodological developments to approximate reality requires the researcher to take more decisions: Does she consider two or three different modes of travel? Does she model fast or slow distance decay? How long does she believe are rural populations willing to travel more than urban populations? Does she consider temporal variation only in travel time or also in demand intensity? This additional uncertainty should be taken into consideration when dealing with increasingly complex gravity models.

*Fitting context:* Some methodological developments have been created against the background of a specific healthcare system or context. These are methods that model selective population-provider pairings that are not (only) dependent on spatial interaction (i.e., subgroup-specific access measures). They reflect context-specific limitations in provider choice. Methodological developments have been proposed to integrate patient referral within a hierarchical healthcare system with gate-keeping functions (e.g., [48, 69]); to depict subgroup-specific access reflecting a language or ethnicity match [47], and spatial access for patients of specific insurance plans [49]. Such methodological developments can prove useful in research practice, however, may not be easily transferable or relevant across healthcare systems.

*Correcting methodology:* Several studies have unveiled unintended flawed implications of earlier methods and changed the methodology to overcome those implicit limitations. Four conceptual issues were addressed with the studies attempting to correct methodology: Firstly, 2SFCA-implicit demand over-estimation (stemming from counting full populations to the potential demand at multiple provider sites) was rectified by allocating demand using a selection weight mimicking provider competition [39]. Secondly, Delamater [45] showed that standard 2SFCA methods implicitly relied on relative distance friction only. An additional global distance decay function was introduced to correct the implicit disregard of absolute distance friction. Thirdly, it was shown that the spatial access scores of gravity models vary strongly with the specification of the distance friction parameter [38]. Since there is uncertainty about a true value (i.e., representing real-world travel preferences), this translates into uncertainty regarding the spatial access measure. Wan, Zhan et al. [38] offered a remedy by computing a spatial access ratio, i.e., interpreting the spatial access measure in relative terms. This makes the measure stable to different distance impedance parameter definitions. Lastly, Guagliardo et al. [30] addressed the issue of circularity that is inherent to the family of 2SFCA measures. Provider availability is adjusted according to the potential demand of the surrounding population; however, one could argue that this potential demand first must be adjusted for the potential availability of surrounding providers, leading to a circular argument. The KD method avoids circularity by overlaying a provider and a population density layer.

Moving forward, we believe that efforts of bringing the methodological developments under one roof will be particularly valuable. In the past, Wang [21] for example has unified the developments on distance decay within the generalized 2SFCA framework. Such work applied to the wider family of gravity model-type measures would be beneficial for depicting the breadth and depth of methodological developments and understanding them as facets of a common spatial access measurement framework. Relatedly, we need critical reflection and assessment of the practical implications of the methodological developments. Several studies have conducted comparative analyses, e.g., to evaluate implications of different distance decay specifications, distance friction parameters, or other methodological choices (e.g., [23, 38, 46, 62]). Such efforts can help us judge which methodological choices require particular care and deliberation. A comprehensive evaluation spanning the methodological developments presented in this study is thus indicated to identify the contribution of various methodological choices to changes in measure outcomes.

#### Review step 2: application studies

While it may seem striking at first to see the difference in application frequency of 2SFCA-type measures compared to base gravity model or Kernel Density approaches, there are multiple factors explaining this resonance within applied health research. Technically, the basic 2SFCA method is a special form of the base gravity model which disregards locations beyond a certain threshold and assumes equal access to all locations within that threshold. That is, through the catchment areas, a cutoff is introduced. This has two main advantages over the base gravity model: First, it makes intuitive sense to simply omit locations that are far away, as those are unlikely to be visited. Second, introducing a threshold in the form of catchment areas is computationally much less intensive. Moreover, as compared to the KD method, it has been shown that 2SFCA has a clear advantage of retaining the total number of providers within a system, while KD is weaker in that regard, especially in edge areas [29].

Among methodological developments within the 2SFCA family, the application of *distance decay within the catchment area* is widely used. We believe that the intuition of distance decay within a catchment area is convincing and makes the spatial access measure more realistic resulting in such wide usage in health research. In addition, implementing a distance decay function in a 2SFCA framework is relatively easy conceptually and computationally. *Variable catchment area sizes* have seen moderate uptake within the 2SFCA method family. At the same time, more than half of 2SFCA type measures were applied to study areas with a mix of urban and rural regions. This means that many measures are computed for areas with varying degrees of urbanicity without differentiating them in terms of catchment sizes. Luo and Whippo [13] have shown that spatial access is overestimated in urban and underestimated in rural areas when uniform catchment areas are assumed. However, it remains unclear how to best define the catchments that vary between urban and rural areas or between general and specialized care providers as catchment area thresholds are essentially arbitrary. The *outcome unit modification* of 2SFCA type measures (spatial access ratio) to make it interpretable in relative terms has not been implemented widely. While it was shown that using a relative measure makes the indicator less sensitive to the travel friction coefficient specification [38], it is not appropriate in all cases. As the outcome unit cannot be interpreted in absolute terms anymore, statements on the number of available providers per population are not possible. Therefore, the measure allows identifying high- and low-access areas and comparing areas within a healthcare system. For healthcare planning however, a relative access measure is hardly useful. Next, we observed moderate uptake of methodological developments correcting the overestimation of demand by incorporating *provider competition*. Generally, the inherent overestimation of demand should be of concern for all 2SFCA type measures. Yet, the majority of 2SFCA type measures did not consider that. Methodological developments of this category do not require additional data but make intuition and computation more complex which might impede wide use. Using a *local and global distance decay function* to consider both relative and absolute distances was not commonly implemented in applied research. This methodological development offers a remedy to the 2SFCA-inherent issue of implicitly only modeling relative distances within a catchment area [45]. There are no additional data requirements for implementing this approach. However, the intuition at work here is not as straightforward as, for example, is the case for distance decay within the catchment area, possibly preventing its use across the board. Almost none of the 2SFCA-type measures applied *a subgroup-specific access measure*. Arguably, such methodological developments are context-specific and might not be easily transferable across healthcare contexts. While many studies proposed methodological developments to account for *multiple transportation modes*, few studies applied such methods. There might be several reasons for this. Firstly, additional data (mode-specific transport users, mode-specific distances) is required to compute a multi-mode 2SFCA. Secondly, the computation relies on somewhat arbitrary decisions (e.g., which modes of transportation to include and whether catchment sizes should vary by mode of transport). Thirdly, multiple transport modes imply additional computational complexity. Lastly, some application studies incorporated *time-dependent access*, explicitly modeling temporal variation in either travel time, demand, or supply. This allows researchers to model a time-dependent distribution of access at a population location rather than a single point estimate. As this is the most recent type of development, it is not yet widely applied. However, it is expected that the share of studies incorporating a temporal perspective will increase in the future.

More than half of the application studies followed a descriptive purpose. Increasingly, spatial access measures are also used as explanatory factors in observational studies. It seems that knowledge about and computation of gravity models has become sufficiently widespread that these gravity models are commonly used as spatial access indicators in applied health research. However, there is room for improvement when it comes to the routine application of gravity models. Almost all studies generated their spatial access indicator "from scratch" for the purpose of the study. Only rarely did studies rely on secondary data using previously computed spatial access measures. The exception lies in the French healthcare context, in which studies resorted to the same database providing a "ready-made" spatial access measure. That goes to show that if gravity model measures are easily available, e.g., provided by a healthcare authority or a research institute, there are good chances of them being used in applied health research. We believe this to be potential yet to harness. Relatedly, methodological developments that we had categorized as corrections of the 2SFCA methodology have not been implemented across the board. We believe this to be rooted in the additional complexity of the measures as well as less straightforward intuition. Generally, it seems that methodological developments that are intuitive to understand, do not have additional data requirements, and do not have great impacts on computational complexity tend to be applied more frequently. While the intuition of these methodological developments can be challenging to grasp, we believe that they are valuable advancements in the family of gravity models as they unveil and fix implicit flawed assumptions. We argue that researchers should apply such methodological corrections or instead transparently state and acknowledge the limitations of standard 2SFCA measures regarding demand and supply allocation, relative and absolute distance friction, distance friction parameter uncertainty, and circularity issues. To foster the use of gravity models in research practice in general and the application of what we termed methodological corrections in particular, further facilitating efforts are needed. There are multiple software solutions and coding packages available for computing some of the most popular spatial access gravity models (e.g., [70, 71]). Expanding this, alongside a conceptual unification within a common framework, a practical unification bringing all the methodological developments presented in this study together, e.g., within a software allowing for customizable methodological choice as well as parameter setting, will be useful for research practice.

### Strengths and limitations

Our study has some limitations. We focused on spatial access to healthcare in our review, thus incorporating literature from the fields of health geography, health services research, health economics, and public health. There may be studies on gravity model developments from other disciplines that we missed. Additionally, our search strategy to identify applications relied on forward citation search based on methodological studies identified in the previous step. If we happened to miss certain studies proposing methodological developments, we might have also missed application studies that cited those. We believe this to not be of strong concern, however, as most empirical studies cite not only the exact method that was applied, but also basic literature on the topic, which in turn means that they would turn up in the search results with our strategy, nonetheless.

The major strengths of our review are its two-step procedure and the depth and scope of analysis. With the two-step procedure, we were able to identify methodological developments and their empirical applications in one study. We could thus describe major developments in the field of potential spatial healthcare access measurement and in addition deliver insights into how these measures are used in research practice. Incorporating results from over 300 empirical studies allowed us to describe gravity model applications in a comprehensive manner. We believe that this large undertaking provides researchers with a valuable overview of the existing methods, their characteristics, strengths, and drawbacks.

## Conclusions

Based on a gravity model which integrated supply and demand as well as accessibility and availability dimensions [10], two major conceptual developments emerged: The Two-Step Floating Catchment Area (2SFCA) method and the Kernel Density (KD) method. Virtually all other methodological developments that were put forward evolved from the 2SFCA method, thus forming a family of 2SFCA-type methods. Within this 2SFCA method family, novelty categories were identified: Distance decay within the catchment area, variable catchment area sizes, outcome unit modification, provider competition, local and global distance decay, subgroup-specific access, multiple transportation modes, and time-dependent access. The 2SFCA method family was commonly used in research practice: Empirical studies almost exclusively applied methods from the 2SFCA family for measuring spatial healthcare access in a gravity model framework. Among the 2SFCA-type application cases, distance decay within the catchment area was a methodological development widely implemented. Variable catchment area sizes and selection probability to account for provider competition were frequently integrated into spatial access measurement as well. However, the use of the basic 2SFCA method remains common.

Most methodological developments served the purpose of approximating reality by integrating assumptions about real-world conditions, such as higher distance tolerance in rural areas, or varying travel times by mode of transportation. A small share of methodological developments was devoted to making context-specific adaptations. A third group of methodological developments was geared towards correcting 2SFCA-inherent flaws, such as the overestimation of demand. Methodological developments that are conceptually straightforward and simple to implement have caught on widely in empirical application studies, while developments designed to correct the methodology are not used across the board. Most empirical studies using a gravity model were descriptive studies, outlining the spatial access to health services in an area. Increasingly, gravity model measures also served as potential explanatory factors for health outcomes.

Much potential remains in the secondary use of previously computed spatial access measures to further foster their application in research practice. Moreover, wider application of developments designed to rectify implicit methodological flaws would be desirable. To that end, future research should aim for a conceptual and practical unification of the here presented methodological developments under one roof.

### Supplementary Information

**Additional file 1.** Search strategy (Appendix A) and methodological developments (Appendix B: Table B1).

**Additional file 2.** Supporting data: Application studies.

## Data Availability

The datasets used and/or analyzed during the current study are included in the additional information files.

## Abbreviations

2SFCA: Two-step floating catchment area
3SFCA: Three-step floating catchment area
E2SFCA: Enhanced two-step floating catchment area
KD: Kernel density
M2SFCA: Modified two-step floating catchment area
MM-2SFCA: Multi-mode two-step floating catchment area
SPAR: Spatial access ratio
V2SFCA: Variable two-step floating catchment area

## Footnotes

1 All of the studies concerned were carried out in the French context, using the so-called "Localized Potential Access" indicators which were computed by the *Direction de la recherche, des études, de l’évaluation et des statistiques* [81] and made available for research. The E2SFCA methodology was implemented for generating the access indicators. Indicators were computed for several professions within primary care.

2 It is noted here that there were several studies applying a gravity model method without considering the population dimension, i.e., omitting potential demand and thus only accounting for supply density. Equally, several studies used Kernel Density method to model provider density, however not overlaying that with the population density. These application studies were excluded as considering the demand dimension was one of the inclusion criteria.

## References

[1] Levesque JF, Harris MF, Russell G (2013). Patient-centred access to health care: conceptualising access at the interface of health systems and populations. Int J Equity Health..

[2] Rosano A, Loha CA, Falvo R, van der Zee J, Ricciardi W, Guasticchi G (2013). The relationship between avoidable hospitalization and accessibility to primary care: a systematic review. Eur J Public Health..

[3] WHO. Everybody’s business—strengthening health systems to improve health outcomes. WHO’s framework for action. Geneva, Switzerland: World Health Organization; 2007.

[4] Joseph AE, Philips DR. Accessibility and utilization: geographical perspectives on health care delivery. 1984.

[5] Delamater PL, Messina JP, Shortridge AM, Grady SC (2012). Measuring geographic access to health care: raster and network-based methods. Int J Health Geogr..

[6] Penchansky R, Thomas JW (1981). The concept of access: definition and relationship to consumer satisfaction. Med Care.

[7] Khan AA (1992). An integrated approach to measuring potential spatial access to health care services. Socioecon Plann Sci.

[8] Guagliardo MF (2004). Spatial accessibility of primary care: concepts, methods and challenges. Int J Health Geogr.

[9] McLafferty S (2020). Place and quantitative methods: critical directions in quantitative approaches to health and place. Health Place.

[10] Joseph AE, Bantock PR (1982). Measuring potential physical accessibility to general practitioners in rural areas: a method and case study. Soc Sci Med.

[11] Luo W, Wang F (2003). Measures of spatial accessibility to health care in a GIS environment: synthesis and a case study in the Chicago region. Environ Plan B Plan Des.

[12] Luo W, Qi Y (2009). An enhanced two-step floating catchment area (E2SFCA) method for measuring spatial accessibility to primary care physicians. Health Place.

[13] Luo W, Whippo T (2012). Variable catchment sizes for the two-step floating catchment area (2SFCA) method. Health Place.

[14] Mao L, Nekorchuk D (2013). Measuring spatial accessibility to healthcare for populations with multiple transportation modes. Health Place.

[15] Bauer J, Müller R, Brüggmann D, Groneberg DA (2018). Spatial accessibility of primary care in England: a cross-sectional study using a floating catchment area method. Health Serv Res.

[16] Wang L (2011). Analysing spatial accessibility to health care: a case study of access by different immigrant groups to primary care physicians in Toronto. Ann GIS.

[17] Gao F, Kihal W, Le Meur N, Souris M, Deguen S (2016). Assessment of the spatial accessibility to health professionals at French census block level. Int J Equity Health..

[18] McLafferty S, Wang F, Luo L, Butler J (2011). Rural–urban inequalities in late-stage breast cancer: spatial and social dimensions of risk and access. Environ Plan B Plan Des..

[19] Delamater PL, Messina JP, Grady SC, WinklerPrins V, Shortridge AM (2013). Do more hospital beds lead to higher hospitalization rates? A spatial examination of Roemer’s Law. PLoS ONE.

[20] Wan N, Zhan FB, Zou B, Wilson JG (2013). Spatial access to health care services and disparities in colorectal cancer stage at diagnosis in Texas. Prof Geogr..

[21] Wang F (2012). Measurement, optimization, and impact of health care accessibility: a methodological review. Ann Assoc Am Geogr.

[22] Vo A, Plachkinova M, Bhaskar R. Assessing healthcare accessibility algorithms: a comprehensive investigation of two-step floating catchment methodologies family. 2015 Am Conf Inf Syst AMCIS 2015. 2015.

[23] Chen X, Jia P (2019). A comparative analysis of accessibility measures by the two-step floating catchment area (2SFCA) method. Int J Geogr Inf Sci.

[24] Apparicio P, Gelb J, Dubé AS, Kingham S, Gauvin L, Robitaille É (2017). The approaches to measuring the potential spatial access to urban health services revisited: distance types and aggregation-error issues. Int J Health Geogr.

[25] Bivoltsis A, Cervigni E, Trapp G, Knuiman M, Hooper P, Ambrosini GL (2018). Food environments and dietary intakes among adults: does the type of spatial exposure measurement matter? A systematic review. Int J Health Geogr.

[26] Wood SM, Alston L, Beks H, Mc Namara K, Coffee NT, Clark RA (2023). The application of spatial measures to analyse health service accessibility in Australia: a systematic review and recommendations for future practice. BMC Health Serv Res.

[27] Page MJ, McKenzie JE, Bossuyt PM, Boutron I, Hoffmann TC, Mulrow CD (2021). The PRISMA 2020 statement: an updated guideline for reporting systematic reviews. BMJ.

[28] Vo A, Plachkinova M, Bhaskar R. Assessing healthcare accessibility algorithms: a comprehensive investigation of two-step floating catchment methodologies family. AMCIS 2015 Proceedings. 2015;17:1–12.

[29] Yang DH, Goerge R, Mullner R (2006). Comparing GIS-based methods of measuring spatial accessibility to health services. J Med Syst.

[30] Guagliardo MF, Ronzio CR, Cheung I, Chacko E, Joseph JG (2004). Physician accessibility: an urban case study of pediatric providers. Health Place.

[31] Siegel M, Koller D, Vogt V, Sundmacher L (2016). Developing a composite index of spatial accessibility across different health care sectors: a German example. Health Policy (New York).

[32] Schuurman N, Berube M, Crooks VA (2010). Measuring potential spatial access to primary health care physicians using a modified gravity model. Can Geogr Can.

[33] Jin M, Liu L, Tong D, Gong Y, Liu Y (2019). Evaluating the spatial accessibility and distribution balance of multi-level medical service facilities. Int J Environ Res Public Health.

[34] Tao Z, Cheng Y, Du S, Feng L, Wang S (2020). Accessibility to delivery care in Hubei Province, China. Soc Sci Med.

[35] McGrail MR, Humphreys JS (2014). Measuring spatial accessibility to primary health care services: utilising dynamic catchment sizes. Appl Geogr.

[36] Kim Y, Byon YJ, Yeo H (2018). Enhancing healthcare accessibility measurements using GIS: a case study in Seoul. Korea PLoS One.

[37] Tao Z, Cheng Y, Zheng Q, Li G (2018). Measuring spatial accessibility to healthcare services with constraint of administrative boundary: a case study of Yanqing District, Beijing, China. Int J Equity Health.

[38] Wan N, Zhan FB, Zou B, Chow E (2012). A relative spatial access assessment approach for analyzing potential spatial access to colorectal cancer services in Texas. Appl Geogr.

[39] Wan N, Zou B, Sternberg T (2012). A three-step floating catchment area method for analyzing spatial access to health services. Int J Geogr Inf Sci.

[40] Luo J (2014). Integrating the huff model and floating catchment area methods to analyze spatial access to healthcare services. Trans GIS.

[41] Jang H (2021). A model for measuring healthcare accessibility using the behavior of demand: a conditional logit model-based floating catchment area method. BMC Health Serv Res.

[42] Shen Z, Gao G, Wang Z (2021). Accessibility assessment of prehospital emergency medical services considering supply-demand differences. J Adv Transp.

[43] Tang J-H, Chiu Y-H, Chiang P-H, Su M-D, Chan T-C (2017). A flow-based statistical model integrating spatial and nonspatial dimensions to measure healthcare access. Health Place.

[44] Paez A, Higgins CD, Vivona SF (2019). Demand and level of service inflation in Floating Catchment Area (FCA) methods. PLoS ONE.

[45] Delamater P (2013). Spatial accessibility in suboptimally configured health care systems: a modified two-step floating catchment area (M2SFCA) metric. Health Place.

[46] Bauer J, Groneberg DA (2016). Measuring spatial accessibility of health care providers—introduction of a variable distance decay function within the floating catchment area (FCA) method. PLoS ONE.

[47] Wang L (2007). Immigration, ethnicity, and accessibility to culturally diverse family physicians. Health Place.

[48] Xiao Y, Chen X, Li Q, Jia P, Li L, Chen Z (2021). Towards healthy China 2030: Modeling health care accessibility with patient referral. Soc Sci Med.

[49] Shao Y, Luo W (2022). Supply-demand adjusted two-steps floating catchment area (SDA-2SFCA) model for measuring spatial access to health care. Soc Sci Med.

[50] Langford M, Higgs G, Fry R (2016). Multi-modal two-step floating catchment area analysis of primary health care accessibility. Health Place.

[51] Zhou X, Yu Z, Yuan L, Wang L, Wu C (2020). Measuring accessibility of healthcare facilities for populations with multiple transportation modes considering residential transportation mode choice. ISPRS Int J Geo-Inf.

[52] Ma L, Luo N, Wan T, Hu C, Peng M (2018). An improved healthcare accessibility measure considering the temporal dimension and population demand of different ages. Int J Environ Res Public Health.

[53] Song Y, Tan Y, Song Y, Wu P, Cheng JC, Kim MJ (2018). Spatial and temporal variations of spatial population accessibility to public hospitals: a case study of rural–urban comparison. GIScience Remote Sens..

[54] Xia T, Song X, Zhang H, Song X, Kanasugi H, Shibasaki R (2019). Measuring spatio-temporal accessibility to emergency medical services through big GPS data. Health Place.

[55] Paul J, Edwards E (2019). Temporal availability of public health care in developing countries of the Caribbean: an improved two-step floating catchment area method for estimating spatial accessibility to health care. Int J Health Plann Manage..

[56] Wang F, Luo W (2005). Assessing spatial and nonspatial factors for healthcare access: towards an integrated approach to defining health professional shortage areas. Health Place.

[57] Delamater P, Shortridge AM, Kilcoyne RC (2019). Using floating catchment area (FCA) metrics to predict health care utilization patterns. BMC Health Serv Res.

[58] Zahnd WE, Josey MJ, Schootman M, Eberth JM (2021). Spatial accessibility to colonoscopy and its role in predicting late-stage colorectal cancer. Health Serv Res.

[59] Wan N, Zhan FB, Lu Y, Tiefenbacher JP (2012). Access to healthcare and disparities in colorectal cancer survival in Texas. Health Place.

[60] Langford M, Higgs G (2006). Measuring potential access to primary healthcare services: the influence of alternative spatial representations of population. Prof Geogr.

[61] Gautam S, Li Y, Johnson TG (2014). Do alternative spatial healthcare access measures tell the same story?. GeoJournal.

[62] McGrail MR (2012). Spatial accessibility of primary health care utilising the two step floating catchment area method: an assessment of recent improvements. Int J Health Geogr.

[63] Jumadi J, Fikriyah VN, Hadibasyir HZ, Sunariya MIT, Priyono KD, Setiyadi NA (2022). Spatiotemporal accessibility of COVID-19 healthcare facilities in Jakarta, Indonesia. Sustainability.

[64] Kim K, Kwon K (2022). Time-varying spatial accessibility of primary healthcare services based on spatiotemporal variations in demand, supply, and traffic conditions: a case study of Seoul South Korea. J Transp Health..

[65] Brizan-St. Martin R, Paul J (2022). Evaluating the performance of GIS methodologies for quantifying spatial accessibility to healthcare in Multi-Island Micro States (MIMS). Health Policy Plan.

[66] Xiong Q, Liu Y, Xing L, Wang L, Ding Y, Liu Y (2022). Measuring spatio-temporal disparity of location-based accessibility to emergency medical services. Health Place.

[67] Dai D (2010). Black residential segregation, disparities in spatial access to health care facilities, and late-stage breast cancer diagnosis in metropolitan Detroit. Health Place.

[68] Jamtsho S, Corner R, Dewan A (2015). Spatio-temporal analysis of spatial accessibility to primary health care in Bhutan. ISPRS Int J Geo-Inf.

[69] Yang N, Shen L, Shu T, Liao S, Peng Y, Wang J (2021). An integrative method for analyzing spatial accessibility in the hierarchical diagnosis and treatment system in China. Soc Sci Med.

[70] Zhu H, Wang F, Wang F (2014). Appendix 5B: A toolkit for automated spatial accessibility measures. Quant methods socio-economic appl GIS.

[71] Saxon J, Koschinsky J, Acosta K, Anguiano V, Anselin L, Rey S (2020). An open software environment to make spatial access metrics more accessible.

[72] McGrail MR, Humphreys JS (2009). A new index of access to primary care services in rural areas. Aust N Z J Public Health.

[73] Plachkinova M, Vo A, Bhaskar R, Hilton B (2018). A conceptual framework for quality healthcare accessibility: a scalable approach for big data technologies. Inf Syst Front.

[74] Ni J, Wang J, Rui Y, Qian T (2015). An enhanced variable two-step floating catchment area method for measuring spatial accessibility to residential care facilities in Nanjing. Int J Environ Res Public Health.

[75] Bozorgi P, Eberth JM, Eidson JP, Porter DE (2021). Facility attractiveness and social vulnerability impacts on spatial accessibility to opioid treatment programs in south carolina. Int J Environ Res Public Health.

[76] Matthews KA, Gaglioti AH, Holt JB, Wheaton AG, Croft JB (2020). Estimating health service utilization potential using the supply-concentric demand-accumulation spatial availability index: a pulmonary rehabilitation case study. Int J Health Geogr.

[77] Polzin P, Borges J, Coelho A (2014). An extended kernel density two-step floating catchment area method to analyze access to health care. Environ Plann B Plann Des.

[78] Ni J, Liang M, Lin Y, Wu Y, Wang C (2019). Multi-mode two-step floating catchment area (2SFCA) method to measure the potential spatial accessibility of healthcare services. ISPRS Int J Geo-Inf.

[79] Tao Z, Cheng Y (2019). Modelling the spatial accessibility of the elderly to healthcare services in Beijing, China. Environ Plan B Urban Anal City Sci.

[80] Xing J, Ng ST (2022). Analyzing spatiotemporal accessibility patterns to tertiary healthcare services by integrating total travel cost into an improved E3SFCA method in Changsha, China. Cities.

[81] Barlet M, Coldefy M, Collin C, Lucas-Gabrielli V. L’Accessibilité potentielle localisée (APL) : une nouvelle mesure de l’accessibilité aux soins appliquée aux médecins généralistes libéraux en France. Série Études Rech Dir la Rech des études l’évaluation des Stat. 2012.

