# Supplementary material for: Gravity models for potential spatial healthcare access measurement: a systematic methodological review
Source: Int J Health Geogr. 2023 Dec 1;22:34. doi: 10.1186/s12942-023-00358-z (PMC10693160; doi:10.1186/s12942-023-00358-z)
Supplement: Supplementary file 1 — Additional file 1. Search strategy (Appendix A) and methodological developments (Appendix B: Table B1). [file 12942_2023_358_MOESM1_ESM.pdf]

APPENDIX A

SEARCH STRATEGY

Search terms for application in databases:

Method concept	Keyword concepts	Search term
Floating catchment area methods	• Floating catchment area<br>• Healthcare	("floating catchment area" OR "floating catchment areas") AND ((healthcare OR (health AND care)) OR ((health OR care) AND service*) OR ((health OR care) AND provider*))
Gravity models	• Gravity / Kernel<br>• Access<br>• Spatial<br>• Healthcare	(kernel OR "gravity model") AND access* AND (spatial* OR geograph*) AND ((healthcare OR (health AND care)) OR ((health OR care) AND service*) OR ((health OR care) AND provider*))
Technical method description	• Raster / grid<br>• Access<br>• Spatial<br>• Indicator<br>• Healthcare	(raster* OR grid* OR vector* OR "network-based") AND access* AND (spatial* OR geograph*) AND (measure* OR indicator* OR index) AND ((healthcare OR (health AND care)) OR ((health OR care) AND service*) OR ((health OR care) AND provider*))
Spatial access indices	• Spatial access<br>• Indicator<br>• Healthcare	("spatial access" OR "spatial accessibility" OR "geographic access" OR "geographical access" OR "geographic accessibility" OR "geographical accessibility") AND (measure* OR indicator* OR index) AND ((healthcare OR (health AND care)) OR ((health OR care) AND service*) OR ((health OR care) AND provider*))
Healthcare access indices	• Healthcare access<br>• Spatial<br>• Indicator	("health access" OR "healthcare access" OR "care access" OR "care accessibility" OR "healthcare accessibility") AND (spatial* OR geograph*) AND (measure* OR indicator* OR index)

Keyword clouds as base for search terms:

Keyword concept	Keyword cloud
• Floating catchment area	("floating catchment area" OR "floating catchment areas")
• Kernel / Gravity	(kernel OR "gravity model")
• Raster / Grid	(raster* OR grid* OR vector* OR "network-based")
• Spatial access	("spatial access" OR "spatial accessibility" OR "geographic access" OR "geographical access" OR "geographic accessibility" OR "geographical accessibility")
• Healthcare access	("health access" OR "healthcare access" OR "care access" OR "care accessibility" OR "healthcare accessibility")
• Indicator	(measure* OR indicator* OR index)
• Access	(access*)
• Spatial	(spatial* OR geograph*)
• Healthcare	((healthcare OR (health AND care)) OR<br>((health OR care) AND service*) OR<br>((health OR care) AND provider*))

Combined search strategy:

```
(
    (
        (
            (
                (
                    (
                        (
                        (raster* OR grid* OR vector* OR "network-based")
                        OR
                        ("health access" OR "healthcare access" OR "care
                        access" OR "care accessibility" OR "healthcare
                        accessibility")
                        OR
                        ("spatial access" OR "spatial accessibility" OR
                        "geographic access" OR "geographical access" OR
                        "geographic accessibility" OR "geographical
                        accessibility")
                        )
                    AND
                    (measure* OR indicator* OR index)
                    )
                OR
                (kernel OR "gravity model")
                )
            AND
            (access*)
            )
        AND
        (spatial* OR geograph*)
        )
    OR
    ("floating catchment area" OR "floating catchment areas")
    )
AND
    (
        (healthcare OR (health AND care))       OR
        ((health OR care) AND service*)          OR
        ((health OR care) AND provider*)
    )
)
```

Note: The keyword cloud regarding the "healthcare" concept is only applied in the multidisciplinary databases *Web of Science Core Collection* and *Scopus*. The subject-specific databases *MEDLINE*, *EMBASE*, and *CINAHL* are searched without this restriction.

APPENDIX B

Table B1: Methodological Developments

Authors	Year	Title	Novelty Type	Methodological development
Luo, W.; Wang, F.	2003	Measures of spatial accessibility to health care in a GIS environment: Synthesis and a case study in the Chicago region	Gravity Model: Conceptual	• **2SFCA: Two-Step Floating Catchment Area method**<br>• Floating catchment areas for determining access<br>• Only locations within pre-defined catchment area radius are considered
Guagliardo, M. F.; Ronzio, C. R.; Cheung, I.; Chacko, E.; Joseph, J. G.	2004	Physician accessibility: An urban case study of pediatric providers	Gravity Model: Conceptual	• **Kernel Density Method**<br>• Provider and population density layers are created using Kernel Density function<br>• Layers overlaid to create provider-to-population ratios
Khan, A. A.	1992	An integrated approach to measuring potential spatial access to health care services	Gravity Model: Adaptation	• Potential availability factor instead of potential demand<br>• Multiple transportation modes weighted by transport use share<br>• Relative access measure
Siegel, M.; Koller, D.; Vogt, V.; Sundmacher, L.	2016	Developing a composite index of spatial accessibility across different health care sectors: A German example	Gravity Model: Adaptation	• Composite access index for entire healthcare system<br>• Aggregated using provider-specific weights

Authors	Year	Title	Novelty Type	Methodological development
Luo, W.; Qi, Y.	2009	An enhanced two-step floating catchment area (E2SFCA) method for measuring spatial accessibility to primary care physicians	2SFCA: Distance Decay within Catchment Area	• **E2SFCA: Enhanced Two-Step Floating Catchment Area method**<br>• **Introduction of distance decay within the catchment area**<br>• Stepwise discrete distance decay<br>• Three travel time zones within catchment area with fixed Gaussian-based weight for each zone
McGrail, M. R.; Humphreys, J. S.	2009	The index of rural access: An innovative integrated approach for measuring primary care access	2SFCA: Distance Decay	• Hybrid distance decay<br>• Two travel time zones within catchment area with no decay in first zone and continuous linear decay function in second zone
Dai, D.	2010	Black residential segregation, disparities in spatial access to healthcare facilities, and late-stage breast cancer diagnosis in metropolitan Detroit	2SFCA: Distance Decay	• Continuous distance decay<br>• Continuous Gaussian decay function within catchment area
Schuurman, N.; Berube, M.; Crooks, V. A.	2010	Measuring potential spatial access to primary health care physicians using a modified gravity model	2SFCA: Distance Decay	• Hybrid distance decay<br>• Two travel time zones within catchment area with no decay in first zone and negative power decay function in second zone
Plachkinova, M.; Vo, A.; Bhaskar, R.; Hilton, B.	2018	A conceptual framework for quality healthcare accessibility: a scalable approach for big data technologies	2SFCA: Distance Decay	• Additive distance decay<br>• Additive travel friction: average travel friction within catchment area added to provider-to-population ratio

Authors	Year	Title	Novelty Type	Methodological development
Jin, M.; Liu, L.; Tong, D.; Gong, Y.; Liu, Y.	2019	Evaluating the spatial accessibility and distribution balance of multi-level medical service facilities	2SFCA: Distance Decay	• Continuous distance decay<br>• Continuous negative power decay function within catchment area<br>• Decay parameter conditional on hospital specialization degree
Tao, Z.; Cheng, Y.; Du, S.; Feng, L.; Wang, S.	2020	Accessibility to delivery care in Hubei Province, China	2SFCA: Distance Decay	• Continuous distance decay<br>• Continuous negative power decay function within catchment area<br>• Decay parameter conditional on hospital specialization degree and region type
Luo, W.; Whippo, T.	2012	Variable catchment sizes for the two-step floating catchment area (2SFCA) method	2SFCA: Variable Catchment Area Sizes	**• V2SFCA: Variable Two-Step Floating Catchment Area method**<br>**• Introduction of variable catchment sizes**<br>• Dynamic catchment size determination: Catchment size increased until base provider-to-population ratio is met
McGrail, M. R.; Humphreys, J. S.	2014	Measuring spatial accessibility to primary health care services: Utilising dynamic catchment sizes	2SFCA: Catchment Area	• Static catchment size determination: Different (pre-defined) catchment sizes conditional on region's degree of rurality
Jamtsho, S.; Corner, R.; Dewan, A.	2015	Spatio-temporal analysis of spatial accessibility to primary health care in Bhutan	2SFCA: Catchment Area	• Dynamic catchment size determination: Catchment size conditional on two nearest neighboring providers – defined by distance to second nearest provider
Ni, J.; Wang, J.; Rui, Y.; Qian, T.	2015	An enhanced variable two-step floating catchment area method for measuring spatial accessibility to residential care facilities in Nanjing	2SFCA: Catchment Area	• Dynamic catchment size determination: Catchment size increased until base provider-to-population ratio is met<br>• Additionally, enforces strict overlap of provider and population catchment areas

Authors	Year	Title	Novelty Type	Methodological development
Kim, Y.; Byon, Y. J.; Yeo, H.	2018	Enhancing healthcare accessibility measurements using GIS: A case study in Seoul, Korea	2SFCA: Catchment Area	• Static catchment size determination: Different (pre-defined) catchment sizes conditional on number of physicians at hospital
Tao, Z.; Cheng, Y.; Zheng, Q.; Li, G.	2018	Measuring spatial accessibility to healthcare services with constraint of administrative boundary: A case study of Yanqing District, Beijing, China	2SFCA: Catchment Area	• Static catchment size determination: Different (pre-defined) catchment sizes conditional degree of hospital specialization<br>• Additionally, only provider / population locations within same administrative area considered
Bozorgi, P.; Eberth, J. M.; Eidson, J. P.; Porter, D. E.	2021	Facility attractiveness and social vulnerability impacts on spatial accessibility to opioid treatment programs in south carolina	2SFCA: Catchment Area	• Static & dynamic catchment size determination: Different catchment size classes conditional on region's degree of urbanization; within size class, catchment size dependent on facility attractivity
Wan, N.; Zhan, F. B.; Zou, B.; Chow, E.	2012	A relative spatial access assessment approach for analyzing potential spatial access to colorectal cancer services in Texas	2SFCA: Outcome Unit Modification	**• SPAR: Spatial Access Ratio**<br>**• Introduction of relative spatial access measure**<br>• Location-specific access score divided by average access score to generate spatial access ratio
Wan, N.; Zou, B.; Sternberg, T.	2012	A three-step floating catchment area method for analyzing spatial access to health services	2SFCA: Provider Competition	**• 3SFCA: 3-Step Floating Catchment Area Method**<br>**• Introduction of provider competition-based choice probability correcting demand over-estimation:** Selection weight for each provider-to-population pair, based on relative distances<br>• Selection weight applied in second step to mimic provider competition and thus correct for over-estimation of demand

Authors	Year	Title	Novelty Type	Methodological development
Luo, J.	2014	Integrating the huff model and floating catchment area methods to analyze spatial access to healthcare services	2SFCA: Provider Competition	• Choice probability: Huff-model based selection weight for each provider-to-population pair<br>• Selection weight incorporates provider capacity and distance decay function in addition to relative distances
Tang, J. H.; Chiu, Y. H.; Chiang, P. H.; Su, M. D.; Chan, T. C.	2017	A flow-based statistical model integrating spatial and nonspatial dimensions to measure healthcare access	2SFCA: Provider Competition	• Choice probability: Rational choice model producing selection weight for each provider-to-population pair<br>• Rational choice model incorporates congestion, distance, capacity
Paez, A.; Higgins, C. D.; Vivona, S. F.	2019	Demand and level of service inflation in Floating Catchment Area (FCA) methods	2SFCA: Provider Competition	• Full proportional allocation of demand instead of choice probability<br>• Population (=demand) is allocated proportionally to providers by standardizing weights to 1; preserving total levels of demand and supply
Matthews, K. A.; Gaglioti, A. H.; Holt, J. B.; Wheaton, A. G.; Croft, J. B.	2020	Estimating health service utilization potential using the supply-concentric demand-accumulation spatial availability index: a pulmonary rehabilitation case study	2SFCA: Provider Competition	• Cumulative demand of all population locations that are closer to provider than population location i<br>• No choice probability
Jang, H.	2021	A model for measuring healthcare accessibility using the behavior of demand: a conditional logit model-based floating catchment area method	2SFCA: Provider Competition	• Choice probability: Conditional logit model producing selection weight for each provider-to-population pair

Authors	Year	Title	Novelty Type	Methodological development
Shen, Z.; Gao, G.; Wang, Z.	2021	Accessibility Assessment of Prehospital Emergency Medical Services considering Supply-Demand Differences	2SFCA: Provider Competition	• Choice probability: Huff-model based selection weight for each provider-to-population pair<br>• Selection weight incorporates provider-to-population ratio and distance decay function
Delamater, P. L.	2013	Spatial accessibility in suboptimally configured health care systems: A modified two-step floating catchment area (M2SFCA) metric	2SFCA: Local & Global Distance Decay	**• M2SFCA: Modified Two-Step Floating Catchment Area method**<br>**• Introduction of additional distance decay function to account for local and global distance decay**<br>• Local and global distance decay considers relative and absolute distances
Bauer, J.; Groneberg, D. A.	2016	Measuring spatial accessibility of health care providers-introduction of a variable distance decay function within the floating catchment area (FCA) method	2SFCA: Local & Global Distance Decay	• Functional forms of local and global distance decay functions differ: adjusted decay function (local decay; relative distances) & constant decay function (global decay; absolute distances)<br>• Adjusted distance decay as function of median & SD distance within catchment area
Wang, L.	2007	Immigration, ethnicity, and accessibility to culturally diverse family physicians	2SFCA: Subgroup-Specific Access	**• Subgroup 2SFCA**<br>**• Introduction of selective provider-population matching**<br>• Sub-group-specific measure for access to matching providers<br>• Overall access score weighted by group-specific provider share divided by group-specific population share in catchment area

Authors	Year	Title	Novelty Type	Methodological development
Xiao, Y.; Chen, X.; Li, Q.; Jia, P.; Li, L.; Chen, Z.	2021	Towards healthy China 2030: Modeling health care accessibility with patient referral	2SFCA: Subgroup-Specific Access	• Patient referral in hierarchical healthcare system<br>• Potential demand on higher provider hierarchy level includes referrals from lower level providers dynamically
Yang, N.; Shen, L.; Shu, T.; Liao, S.; Peng, Y.; Wang, J.	2021	An integrative method for analyzing spatial accessibility in the hierarchical diagnosis and treatment system in China	2SFCA: Subgroup-Specific Access	• Patient referral in hierarchical healthcare system<br>• Potential demand on higher provider hierarchy level includes referrals from lower level providers statically
Shao, Y.; Luo, W.	2022	Supply-demand adjusted two-steps floating catchment area (SDA-2SFCA) model for measuring spatial access to health care	2SFCA: Subgroup-Specific Access	• Insurance-plan specific access scores<br>• Potential demand considers only populations with matching insurance plans; access score considers only providers with matching insurance plans
Mao, L.; Nekorchuk, D.	2013	Measuring spatial accessibility to healthcare for populations with multiple transportation modes	2SFCA: Multiple Transportation Modes	**• MM-2SFCA: Multi-Mode Two-Step Floating Catchment Area method**<br>**• Introduction of multiple transportation modes**<br>• Considering varying shares of mode-specific transport users and varying mode-specific travel times<br>• Final measure is overall access score (not mode-specific)
Polzin, P.; Borges, J.; Coelho, A.	2014	An extended kernel density two-step floating catchment area method to analyze access to health care	2SFCA: Transportation	• Population size weighted by mobility index

Authors	Year	Title	Novelty Type	Methodological development
Langford, M.; Higgs, G.; Fry, R.	2016	Multi-modal two-step floating catchment area analysis of primary health care accessibility	2SFCA: Transportation	• Considering varying shares of mode-specific transport users and varying mode-specific travel times<br>• Final measure are separate access scores for each transportation mode (mode-specific)
Ni, J.; Liang, M.; Lin, Y.; Wu, Y.; Wang, C.	2019	Multi-mode two-step floating catchment area (2SFCA) method to measure the potential spatial accessibility of healthcare services	2SFCA: Transportation	• Multiple transportation modes considered sequentially (several modes per trip)<br>• Choice probability for each mode at each distance range of trip
Tao, Z.; Cheng, Y.	2019	Modelling the spatial accessibility of the elderly to healthcare services in Beijing, China	2SFCA: Transportation	• Considering varying shares of mode-specific transport users; varying mode-specific travel times; varying shares of elderly population<br>• Final measure is access score for elderly population (not mode-specific); accounting for competition with (differently mobile) non-elderly
Zhou, X.; Yu, Z.; Yuan, L.; Wang, L.; Wu, C.	2020	Measuring accessibility of healthcare facilities for populations with multiple transportation modes considering residential transportation mode choice	2SFCA: Transportation	• Travel mode choice probability for each provider-to-population pair (instead of population location-specific mode user share)<br>• Final measure is overall access score (not mode-specific)
Xing, J.; Ng, S. T.	2022	Analyzing spatiotemporal accessibility patterns to tertiary healthcare services by integrating total travel cost into an improved E3SFCA method in Changsha, China	2SFCA: Transportation	• Considering varying shares of mode-specific transport users and varying mode-specific travel times<br>• Mode-specific distance decay function<br>• Final measure are separate access scores for each transportation mode (mode-specific)

Authors	Year	Title	Novelty Type	Methodological development
Ma, L.; Luo, N.; Wan, T.; Hu, C.; Peng, M.	2018	An improved healthcare accessibility measure considering the temporal dimension and population demand of different ages	2SFCA: Time-Dependent Access	• **Spatio-Temporal 2SFCA**<br>• Dynamic (time-varying) access measure<br>• Travel time dependent on time of day due to traffic<br>• Time-varying (real-time) traffic conditions
Song, Y. Z.; Tan, Y.; Song, Y. M.; Wu, P.; Cheng, J. C.P.; Kim, M. J.; Wang, X.	2018	Spatial and temporal variations of spatial population accessibility to public hospitals: a case study of rural-urban comparison	2SFCA: Time-Dependent Access	• Travel time dependent on season due to road conditions<br>• Time-varying (seasonal average) travel speed
Paul, J.; Edwards, E.	2019	Temporal availability of public health care in developing countries of the Caribbean: An improved two-step floating catchment area method for estimating spatial accessibility to health care	2SFCA: Time-Dependent Access	• Healthcare supply dependent on time of day due to provider schedule<br>• Time-varying provider availability
Xia, T.; Song, X.; Zhang, H.; Kanasugi, H.; Shibasaki, R.	2019	Measuring spatio-temporal accessibility to emergency medical services through big GPS data	2SFCA: Time-Dependent Access	• Population size dependent on time of day due to commuting behavior<br>• Time-varying population distribution
